# The autoactivation of human single-chain urokinase-type plasminogen activator (uPA)

**DOI:** 10.1016/j.jbc.2023.105179

**Published:** 2023-08-20

**Authors:** Constanza Torres-Paris, Yueyi Chen, Lufan Xiao, Harriet J. Song, Pingyu Chen, Elizabeth A. Komives

**Affiliations:** Department of Chemistry and Biochemistry, University of California, San Diego, La Jolla, California, USA

**Keywords:** serine protease, hydrogen–deuterium exchange mass spectrometry, plasminogen activation, enzyme processing, plasmin, fibrinolysis, anticoagulation pathway, enzyme dynamics

## Abstract

Most serine proteases are synthesized as inactive zymogens that are activated by cleavage by another protease in a tightly regulated mechanism. The urokinase-type plasminogen activator (uPA) and plasmin cleave and activate each other, constituting a positive feedback loop. How this mutual activation cycle begins has remained a mystery. We used hydrogen deuterium exchange mass spectrometry to characterize the dynamic differences between the inactive single-chain uPA (scuPA) and its active form two-chain uPA (tcuPA). The results show that the C-terminal β-barrel and the area around the new N terminus have significantly reduced dynamics in tcuPA as compared with scuPA. We also show that the zymogen scuPA is inactive but can, upon storage, become active in the absence of external proteases. In addition to plasmin, the tcuPA can activate scuPA by cleavage at K158, a process called autoactivation. Unexpectedly, tcuPA can cleave at position 158 even when this site is mutated. TcuPA can also cleave scuPA after K135 or K136 in the disordered linker, which generates the soluble protease domain of uPA. Plasmin cleaves scuPA exclusively after K158 and at a faster rate than tcuPA. We propose a mechanism by which the uPA receptor dimerization could promote autoactivation of scuPA on cell surfaces. These results resolve long-standing controversies in the literature surrounding the mechanism of uPA activation.

Plasminogen activators catalyze the activation of the zymogen, plasminogen, into the active protease plasmin. Plasmin degrades the fibrin fibrils in blood clots and activates matrix metalloproteinases that contribute to modifying the extracellular matrix promoting tissue remodeling, cell migration, wound healing, and inflammation, among other cellular functions (1, 2). There are two types of plasminogen activators: the tissue-type plasminogen activator and the urokinase-type plasminogen activator (uPA). While tissue-type plasminogen activator has mostly been associated with vascular fibrinolysis, uPA has been proposed to catalyze plasminogen activation in tissue remodeling (3, 4). The uPA has three domains (Fig. 1*A*): an epidermal growth factor (EGF)–like domain and a kringle domain, which together constitute the amino terminal fragment (ATF), and a serine protease domain. The ATF and the serine protease domain are tethered by a 27-residue disordered linker. The uPA is localized in the cell surface through binding of the EGF-like domain to the uPA receptor (uPAR), a membrane-anchored glycoprotein (5). The interaction of the ATF with the uPAR triggers the activation of several signaling cascades, which promote cell adhesion, cell migration, and angiogenesis, among others (4). Strong evidence shows that uPAR dimerizes in cell surfaces and that uPA can bind the uPAR dimer (3, 6, 7, 8).

**Figure 1:**
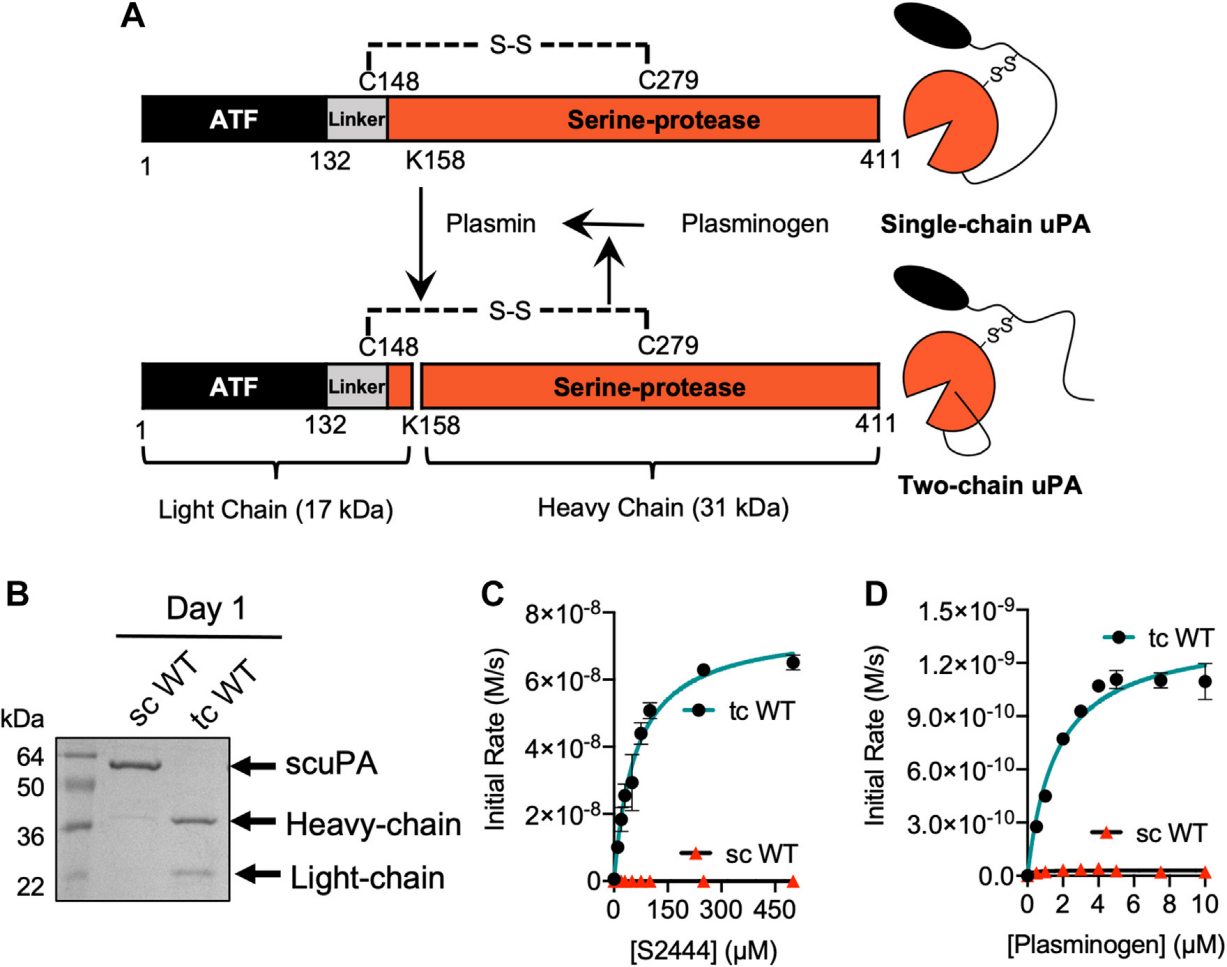
**The zymogen scuPA is inactive and tcuPA is active.***A*, schematic diagram of the positive feedback loop between uPA and plasmin. The scuPA zymogen needs to be cleaved after K158 by plasmin, and plasminogen needs to be cleaved by tcuPA. The two chains of uPA are connected by the disulfide bond between C148(1_CT_) and C279(122_CT_). It is unclear which protease gets activated first. *B*, SDS-PAGE under reducing conditions showing scuPA and tcuPA immediately after purification on day 1. *C*, Michaelis–Menten plot of tcuPA and scuPA (10 nM) cleavage if the chromogenic substrate analog S2444 (10–500 μM). *D*, Michaelis–Menten plot of tcuPA and scuPA (10 nM) cleavage of Glu–plasminogen (0.5–10 μM). Data for each substrate concentration in the Michaelis–Menten curves represent the mean and standard deviation of two technical replicates. scuPA, single-chain uPA; tcuPA, two-chain uPA; uPA, urokinase-type plasminogen activator.

The uPA is synthesized as a single-chain zymogen (single-chain uPA [scuPA]) that can be cleaved between K158(15_CT_), the subscript CT stands for the chymotrypsin numbering) and I159(16_CT_) to yield a two-chain form (two-chain uPA [tcuPA]), where the two polypeptide chains remain linked by a disulfide bond (Fig. 1*A*) (9). Even though it is well established that the maximum activity of uPA is achieved in its two-chain form, it is not clear to what degree scuPA has any activity. Whereas some groups have shown that scuPA is not active (10), others have detected low intrinsic activity (11, 12). Plasmin is the protease that catalyzes the activation of scuPA into tcuPA, whereas uPA converts the zymogen plasminogen into its active form, plasmin, thus constituting a positive feedback loop. It has also been suggested that plasmin can cleave uPA in the disordered linker between K135 and K136 (13, 14, 15, 16). This cleavage is in the linker region of uPA before the disulfide bond that connects the ATF to the protease domain and releases the protease domain from the cell surfaces (17). There are two species of uPA that can be detected in urine: the full-length protein or “high-molecular weight uPA” and a version of uPA that starts at K136 and includes the protease domain, called “low-molecular weight (LMW) uPA” (16, 18). Both high-molecular weight and LMW uPAs are active proteases.

All serine proteases are activated by a proteolytic cleavage event that results in a new N terminus, which inserts into a hydrophobic pocket and forms a salt bridge with an Asp, thus orienting the catalytic triad (19). Only the structure of the protease domain of uPA after plasmin cleavage has been solved (20). It shows that the newly generated N terminus at I159(16_CT_) inserts into a pocket and orients the catalytic triad: residues H204(57_CT_), D255(102_CT_), and S356(195_CT_). The protease domain shows a classic chymotrypsin fold with two perpendicular β-barrels with the catalytic triad between them. As the structure of scuPA has not been solved, it remains unknown if activation generates any structural changes in other parts of the protease domain beyond the catalytic triad. Evidence of structural and dynamical differences between other zymogens and their respective active proteases has been demonstrated by hydrogen–deuterium exchange mass spectrometry (HDX-MS). The fully activated forms of factors VIIa, IXa, and XIa do not show significant changes in deuterium incorporation after cleavage (21, 22, 23), whereas thrombin shows a decrease in the dynamics of one β-barrel and an increase in the other (24). Previous work on the murine uPA protease domain showed that antibody binding changes the protease activity and the dynamics of the loops of uPA (25, 26), but the potential changes in dynamics between scuPA and tcuPA remain unknown.

There is no consensus on how the plasmin–uPA activation feedback loop starts. It has been proposed that the onset of the positive feedback loop can be due to the activation of scuPA by cleavage with several proteases, including cathepsin L, stromelysin-1, nerve growth factor gamma, mast cell tryptase, factor XIIa, kallikrein, cathepsin B, and tumor-associated trypsin (27, 28, 29, 30, 31, 32, 33). All these proteases are less efficient than plasmin in activating scuPA, and their expression is limited to certain tissues, which suggests that the positive feedback loop could have different tissue-dependent activation mechanisms. It has also been shown that chicken uPA is able to autoactivate (34, 35); however, it remains to be characterized whether human scuPA can autoactivate.

In this article, we report the differences in dynamics between scuPA and tcuPA by HDX-MS. We also investigate when and how human scuPA can be autoactivated, defined as the ability of the active form of a zymogen (tcuPA) to activate its own zymogen (scuPA). We show that human tcuPA can cleave scuPA after K158(15_CT_) and activate it; and can also carry out a second self-cleavage in the linker between K135 and K136, which releases the protease domain from the ATF. We also showed that plasmin can only cleave scuPA after K158, and that plasmin-dependent activation of scuPA is faster than tcuPA-dependent activation of uPA. These results demonstrate that autoactivation of human uPA is an important phenomenon that explains prior discrepancies in the literature and explores a possibility of how the positive feedback loop of uPA and plasmin activation might be initiated.

## Results

### Functional and dynamics differences between scuPA and tcuPA

We expressed and purified full-length human urokinase plasminogen activator (hereafter referred to as uPA) from inclusion bodies in *Escherichia coli.* We developed a novel protocol for its refolding and purification that is detailed in the Experimental procedures section, obtaining the single-chain form (hereafter referred to as scuPA). Plasmin was used to convert scuPA into the two-chain form as shown in Figure 1, *A* and *B*. The activity of scuPA and tcuPA was assayed using the small chromogenic substrate analog S2444 (hereafter referred to as amidolytic activity) and the endogenous substrate of uPA, plasminogen. Our new refolding method yielded a version of tcuPA that had equivalent activity to what other authors have published previously for uPA purified from mammalian cells (36) or from inclusion bodies from *E. coli* (37, 38) using other refolding methods (Table 1). We were unable to measure significant activity of scuPA toward either plasminogen or S2444 (Figs. 1, *C* and *D* and S1).

**Table 1:** Our refolded uPA from inclusion bodies is equally active as previously purified uPA

Protein	Substrate	*k*_cat_ (s^−1^)	*K*_*M*_ (μM)	*k*_cat_/*K*_*M*_ (μM^-−^ s^−1^)
uPA from mammalian cells (36)	Plasminogen	0.73	25	0.029
Refolded with Winkler & Blaber's method (37)	S2444	2.3	82	0.028
	Plasminogen	0.14	4.5	0.030
Refolded with novel method (this work)	S2444	8.5 ± 0.2	44 ± 4	0.19 ± 0.02
	Plasminogen	0.16 ± 0.01	5.6 ± 0.6	0.029 ± 0.003

**Figure 2:**
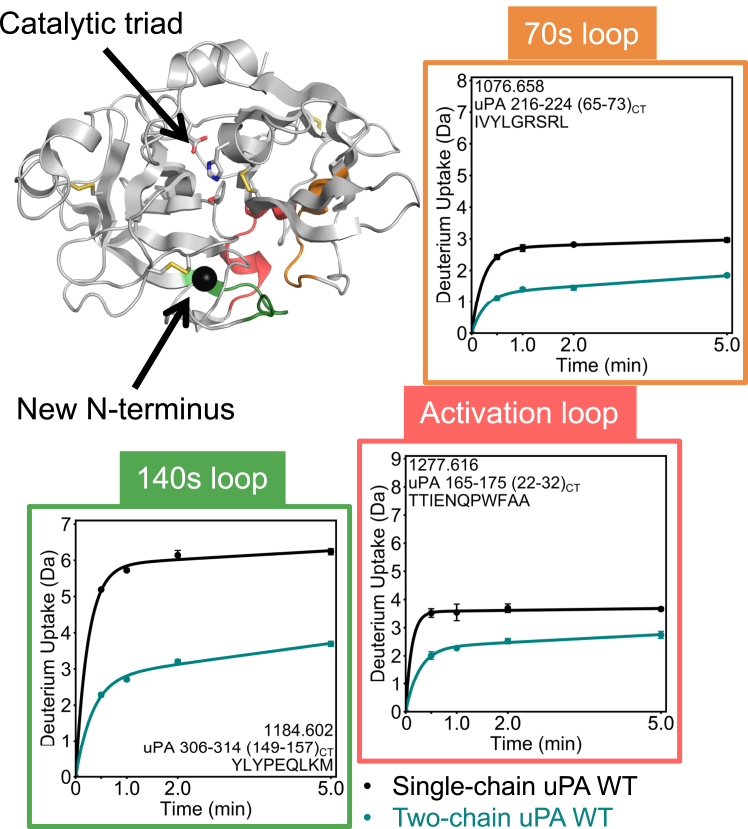
**HDX-MS reveals that plasmin activation reduces the dynamics around the new N-terminal insertion site.** The HDX-MS uptake plots for three regions in the protease domain of tcuPA WT (*teal*) and scuPA WT (*black*) are shown mapped on the crystal structure (Protein Data Bank code: 4DVA). The structural region spanning residues 306 to 314 (149_CT_–157_CT_) is colored *dark green*; the one spanning residues 165 to 175 (22_CT_–32_CT_) is colored *red*; and the one spanning residues 216 to 224 (65_CT_–73_CT_) is colored *orange*. The uptake plots are bordered in the corresponding colors. The side chains of the catalytic triad residues and of the cysteines forming disulfide bonds are shown as *sticks*. The new N terminus is shown as a *black sphere*. Each point in the uptake plots represents the mean and standard deviation of three technical replicates. HDX-MS, hydrogen–deuterium exchange mass spectrometry; scuPA, single-chain uPA; tcuPA, two-chain uPA.

**Figure 3:**
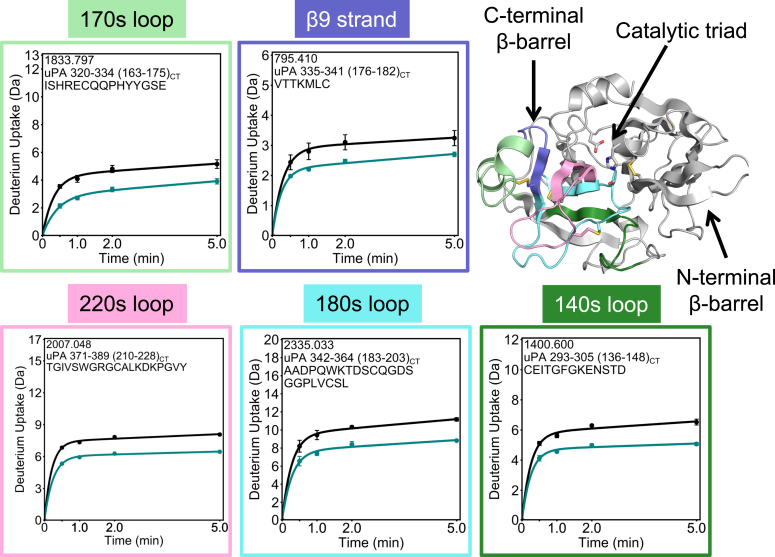
**Activation provokes long-range dynamic allostery in the protease domain of uPA.** The HDX-MS deuterium uptake plots for five regions of the protease domain of tcuPA WT (*teal*) and scuPA WT (*black*) are shown mapped on its crystal structure (Protein Data Bank code: 4DVA). The structural region spanning residues 320 to 334 (163_CT_–175_CT_) is colored *light green*; the one spanning residues 335 to 341 (176_CT_–182_CT_) is colored *purple*; the one spanning residues 371 to 389 (210_CT_–228_CT_) is colored *light pink*; the one spanning residues 342 to 364 (183_CT_–203_CT_) is colored *cyan*, and the one spanning residues 293 to 305 (136_CT_–148_CT_) is colored *dark green*. The uptake plots are bordered in the corresponding colors depicted on the structure. The side chains of the catalytic triad residues and of the cysteines forming disulfide bonds are shown as *sticks*. Each point in the uptake plots represents the mean and standard deviation of three technical replicates. HDX-MS, hydrogen–deuterium exchange mass spectrometry; scuPA, single-chain uPA; tcuPA, two-chain uPA; uPA, urokinase-type plasminogen activator.

To understand what conformational changes might be driving the activation of uPA, both scuPA and tcuPA were analyzed by HDX-MS. Deuterium uptake into scuPA and tcuPA was measured over 0 to 5 min, which is the timescale known to reveal allosteric conformational changes in serine proteases (39, 40). A total of 105 peptides were successfully identified for scuPA and 83 peptides were identified for tcuPA, which covered 94.3% and 84.1% of the sequences, respectively (Table S1). No differences in deuterium incorporation into the ATF were detected for any of the uPA forms (Fig. S2); however, significant differences were observed in the protease domain. Plasmin activation creates a new N terminus (I159(16_CT_)) in the protease domain that inserts into the activation pocket, forming a salt bridge with Asp 355(194_CT_). Consistent with this observation, HDX-MS data showed a significant reduction in deuterium incorporation in tcuPA compared with scuPA into residues 165 to 175 (22_CT_–32_CT_), part of the activation loop (residues 160–175 (17_CT_–32_CT_) (Fig. 2) immediately after the new N terminus in the protease domain. The 140s loop (306–314 [149_CT_–157_CT_]) and the 70s loop (216–224 [65_CT_–73_CT_]), both adjacent to the activation loop, also showed reduced deuterium incorporation in the tcuPA. Residues 293 to 305 (136_CT_–148_CT_) in the 140s loop, residues 320 to 334 (163_CT_–175_CT_) in the 170s loop, residues 342 to 364 (183_CT_–203_CT_) in the 180s loop, and residues 371 to 389 (210_CT_–228_CT_) in the 220s loop all exchanged less in tcuPA as compared with scuPA. No regions of tcuPA showed enhanced deuterium uptake as compared with the same regions in scuPA (Fig. 3). This demonstrates that the plasmin-dependent activation of scuPA into tcuPA leads to a decrease in the dynamics of the protease domain.

### The autoactivation of scuPA during storage

**Figure 4:**
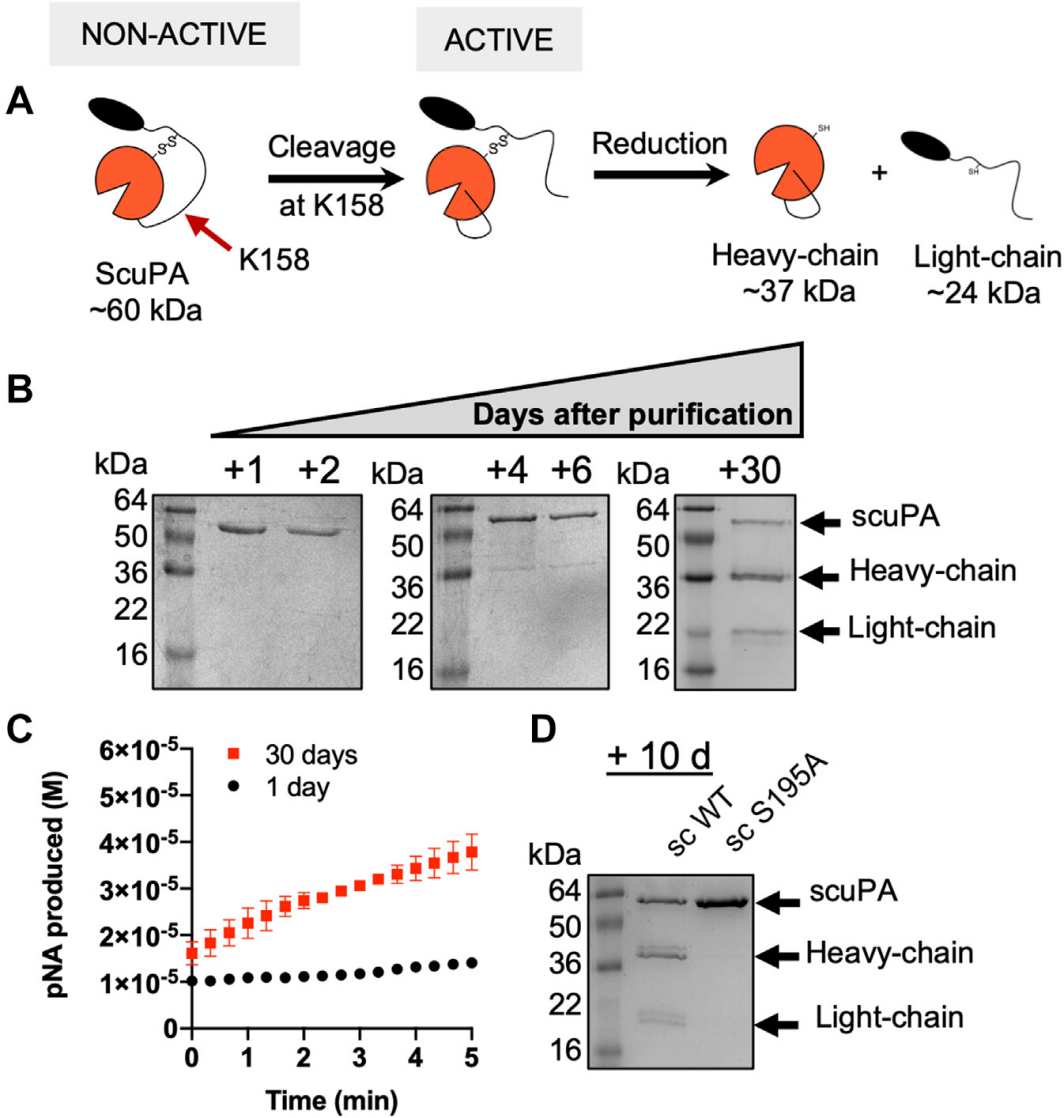
**ScuPA WT becomes cleaved and activated during storage in the absence of plasmin.***A*, *cartoon model* showing where scuPA can be cleaved during autoactivation. *B*, the integrity of scuPA after purification was evaluated by SDS-PAGE. An aliquot of the protein was collected after 1 to 30 days of storage of the protein at 4 °C. The formation of tcuPA was confirmed by the appearance of a faint band with a molecular weight consistent with the heavy chain of uPA. *C*, amidolytic activity of scuPA on day 1 and day 30 of storage at 4 °C using S2444 as substrate. The initial rates of scuPA WT after 1 or 30 days of storage were 8 ± 2 nM/s and 80 ± 20 nM/s, respectively. *D*, SDS-PAGE under reducing conditions of scuPA WT and scuPA S356(195_CT_)A, after 10 days of storage at 4 °C. scuPA, single-chain uPA; tcuPA, two-chain uPA; uPA, urokinase-type plasminogen activator.

Previous authors have shown that chicken scuPA can get cleaved to become active tcuPA *in vitro* within hours in the absence of plasmin and that human uPA cannot become cleaved in the absence of external proteases in that time frame (34, 35). To test whether human scuPA could also get activated in the absence of plasmin over longer periods, freshly purified scuPA was incubated at 4 °C for several days and the cleavage was followed by SDS-PAGE under reducing conditions (Fig. 4*B*). A second protein band of ∼36 kDa apparent molecular weight started to appear after 4 days of incubation and was very evident after 30 days of storage (Fig. 4*B*). A third protein band of ∼24 kDa also became visible after 30 days. A significant increase in amidolytic activity also occurred after 30 days of storage, indicating generation of some form of activated uPA (Fig. 4*C*). Consistent with the activity, in-gel trypsin digestion followed by MS/MS sequencing showed that the ∼36 kDa band corresponded to the heavy chain of uPA and the ∼24 kDa band corresponded to the ATF (Fig. S3).

To confirm that the cleavage was not because of a contaminant protease present, we mutated the catalytic serine, S356(195_CT_) to A to yield a catalytically inactive version of the protease (Fig. S4). The scuPA WT and S356(195_CT_)A mutant were purified and incubated at 4 °C for 10 days, after which cleavage was visualized by SDS-PAGE under reducing conditions (Fig. 4*D*). No cleavage was observed for the catalytically dead mutant, which suggests that this cleavage is dependent on uPA and that no external protease contaminant was present. Combined, these results suggest that human scuPA can also become cleaved in the absence of plasmin.

One possibility to explain the cleavage of scuPA is that scuPA could have some residual intrinsic activity as has been proposed previously (11, 12). It was suggested that K300(143_CT_) in the 140s loop could play a role in explaining some residual activity in scuPA, by inserting into the activation pocket, resulting in intrinsic activity of scuPA, and that the K300(143_CT_)H mutation prevents scuPA from being active (37, 41, 42). The K300(143_CT_)H mutant scuPA became cleaved as much as scuPA WT after 30 days of incubation at 4 °C, suggesting that the K300(143_CT_)H mutation is not enough to stop scuPA from becoming cleaved in storage (Fig. S5).

**Figure 5:**
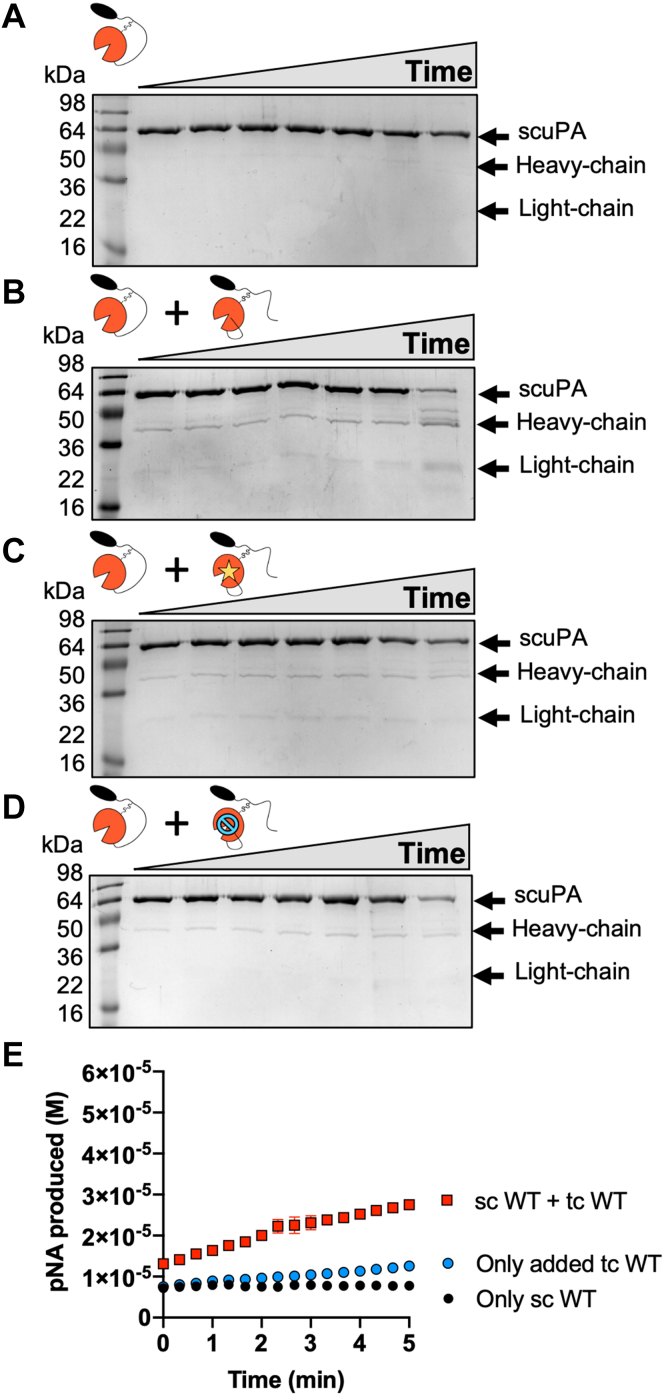
**TcuPA cleaves and activates scuPA.** ScuPA WT (2 μM) was incubated at 37 °C in the absence of any other protease (*A*), in the presence of 0.2 μM tcuPA WT (*B*), in the presence of 0.2 μM tcuPA S356(195_CT_)A (*C*), or in the presence of GGACK-inhibited tcuPA WT (*D*). The reaction products were visualized by SDS-PAGE under reducing conditions. The reaction time points shown correspond to 0, 10 min, 30 min, 1 h, 3 h, 5 h, and 19 h. Note that in (*B*–*D*), the faint bands at 36 and 24 kDa are due to the 0.2 μM added proteases. *E*, the amidolytic activity of the reaction products was measured after 19 h of incubation at 37 °C. The basal activity of the added tcuPA (0.2 μM) was also measured. The graph points represent the average and standard deviation of two technical replicates. GGACK, Glu–Gly–Arg–chloromethyl ketone; scuPA, single-chain uPA; tcuPA, two-chain uPA; uPA, urokinase-type plasminogen activator.

We next tested whether tcuPA could cleave scuPA to an active protease. ScuPA (2 μM) was incubated at 37 °C for up to 19 h, in the presence and absence of 0.2 μM of tcuPA (Fig. 5, *A* and *B*). Under these conditions, scuPA was not cleaved in the absence of another protease (Fig. 5*A*). On the contrary, a very defined pattern of protein bands of lower molecular weight than scuPA were detected after the addition of tcuPA WT (Fig. 5*B*). Some of these bands clearly corresponded to the heavy-chain form of uPA (band at ∼36 kDa) and to the ATF (band at ∼24 kDa). The other bands suggested that tcuPA could cleave scuPA at more than one site. When scuPA WT was incubated in the presence of the catalytically inactive tcuPA S356(195_CT_)A (Fig. 5*C*), or in the presence of tcuPA WT previously inhibited with the uPA-specific inhibitor GGACK (Glu–Gly–Arg–chloromethyl ketone) (Fig. 5*D*), no cleave products were observed. We note that the increase in intensity of the band corresponding to the heavy chain of uPA when scuPA WT was incubated with tcuPA WT correlated with an increase in the amidolytic activity over and above that of the added tcuPA (Fig. 5*E*). These results support the idea that tcuPA can activate its zymogen in the absence of other proteases, as was previously observed for chicken uPA. Our results demonstrate that tcuPA-dependent scuPA activation is an example of autoactivation (43).

### Autoactivation occurs by cleavage after position 158

**Figure 6:**
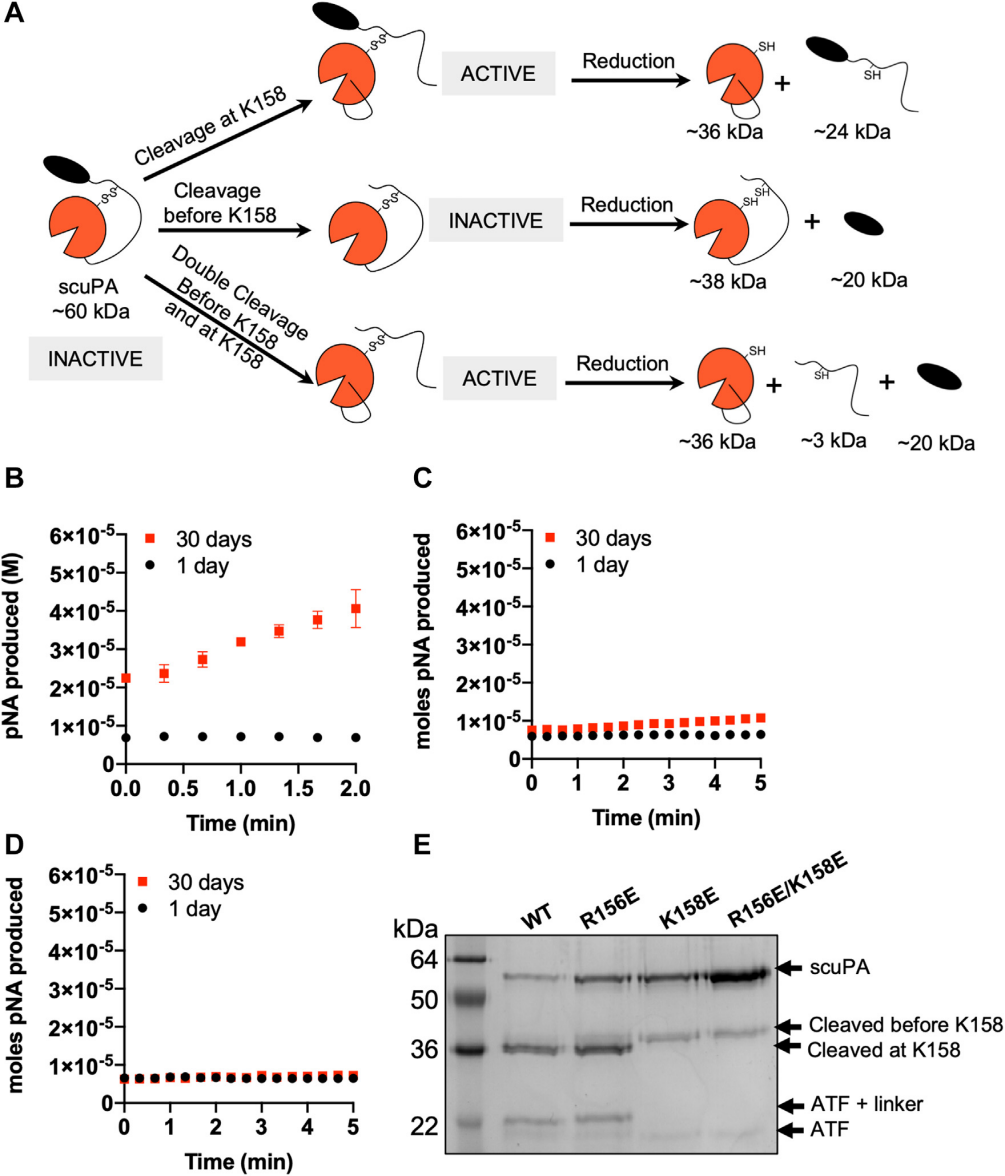
**Autoactivation of scuPA by tcuPA occurs by cleavage at position 158.***A*, *cartoon representation* showing the possible cleavages that happen during autoactivation and the molecular weights of the products they would produce on SDS-PAGE. Only the cleavage after K158 yields an active protease. ScuPA can also be cleaved in the disordered linker before C148; however, that cleavage does not activate the protease. *B*–*D*, amidolytic activity of the scuPA mutant proteins R156E (*B*), K158E (*C*), and R156E/K158E (*D*) on day 1 and day 30 of storage at 4 °C, using S2444 as substrate. The initial rates of scuPA R156E were 0.2 ± 0.6 nM/s (day 1) and 120 ± 50 nM/s (day 30); of scuPA K158E were 3 ± 2 nM/s (day 1) and 9 ± 2 nM/s (day 30); and of scuPA R156E/K158E were 0 ± 1 nM/s (day 1) and 5 ± 1 nM/s (day 30). *E*, SDS-PAGE under reducing conditions showing the scuPA proteins (WT, R156E, K158E, and R156E/K158E) after 30 days of storage at 4 °C. scuPA, single-chain uPA; tcuPA, two-chain uPA; uPA, urokinase-type plasminogen activator.

The only known mechanism of uPA activation is through cleavage between K158(15_CT_) and I159(16_CT_). Previous sequencing of LMW uPA from human urine showed the light chain ending in R156 and the heavy chain starting in K158, which suggests that both cleavages occur under physiological conditions (16). We hypothesized that scuPA could be cleaved after R156 or K158 during storage but that only the protein cleaved after K158 would be active (Fig. 6*A*). To check whether R156 or K158 could both be activation cleavage sites, we mutated R156 and K158 to glutamic acid. The mutant proteins were stored for 30 days at 4 °C, and their autoactivation was tested by comparing the activities on day 1 and day 30 after purification. The activity of the R156E mutant increased as much as the WT protein (Figs. 6*B* and 4C). The K158E mutant protein showed a slight increase in activity after 30 days, which was nine times lower than the increase in the activity of WT (Fig. 6*C*). The double mutant R156E/K158E did not show a significant increase in activity over time (Fig. 6*D*).

To check whether the increase in activity correlated with a specific cleavage pattern, the mutant proteins after 30 days of storage were run on SDS-PAGE under reducing conditions (Fig. 6*E*). These bands were excised, trypsin-digested, and sequenced by MS (Figs. S6 and S7). Because an active protease would form after a cleavage at the beginning of the protease domain, we expected to observe a band corresponding to the heavy chain of the protease domain at ∼36 kDa correlating with an increase of activity, as seen for the WT protein (Fig. 6*A*). Interestingly, storage of the K158E and the R156E/K158E mutants yielded a protein band at ∼38 kDa, which was slightly larger than the band produced from the WT protein and corresponded to the protease domain with some portion of the linker region (Fig. S6). The R156E mutant yielded two bands at ∼36 and ∼38 kDa (Fig. 6*E*). Because of their very closed proximity, it was not possible to identify each individual band by MS. The ∼36 kDa band ran at the same position as the one generated in the WT protein, whereas the other band at ∼38 kDa corresponded to the one present in the K158E and double mutant, suggesting that R156 was cleaved at two sites yielding solely the protease domain or the protease domain with some portion of the linker remaining (Fig. S6). The intensity of the ∼36 kDa band correlated with the intensity of the band at ∼24 kDa, and similarly the ∼38 kDa band correlated with the ∼20 kDa band, suggesting that these are complementary proteolytic products. It was not possible to identify the difference between the ∼24 kDa and ∼20 kDa protein bands by MS, as both contained peptides from the ATF and of other regions of the proteins, which may correspond to a mixture of smaller size proteolytic products, one of them being the ATF (Fig. S7). Overall, these results suggest that when the R156 or the K158 of scuPA are mutated, the protein gets cleaved in another N-terminal location to the K158 to yield a protein of ∼38 kDa. Note that even if this cleavage happens, the newly generated protein is not expected to be active, unless a second cleavage would occur at position 158 (Fig. 6*A*). It is most likely that the increased activity of the R156E mutant results from cleavage at K158 as well. On the contrary, the significantly low activity whenever the K158E mutation is present strongly suggests the importance of K158 mutant for autoactivation.

MS of the trypsinized protein bands was also used to understand why the proteolytically cleaved K158E mutant had activity (Table 2). Trypsin digestion of the ∼36 kDa bands from the K158E and R156E/K158E mutants contained nontryptic peptides cleaved between K158E and I159(16_CT_). Given the high specificity of trypsin (44), the most likely explanation for these results is that uPA can cleave after E158 and that the new N terminus that is generated can still insert and cause formation of an active protease. However, given the lower activity of mutants containing the K158E mutation, the autoactivation is diminished with this mutation.

**Table 2:** Protein sequencing by MS and in-gel trypsin cleavage reveals that scuPA is cleaved after K158 even if is mutated to E

Start	End	Peptide	−logP	Area	Sample
152	158	K.TLRPEFE.I	56.05	2.14E+06	Nonreduced R156E/K158E 60 kDa
159	178	E.IIGGEFTTIENQPW(+15.99)FAAIYR.R	34.5	4.63E+05	Reduced K158E 60 kDa
			18.69	4.60E+05	Reduced R156E/K158E 36 kDa
152	158	E.IIGGEFTTIEN(+0.98)QPW(+15.99)FAAIYR.R	36.59	0	Reduced K158E 60 kDa
			25.38	0	Reduced R156E/K158E 60 kDa

### TcuPA can also cleave scuPA between K135 and K136 in the linker before C148(1_CT_)

**Figure 7:**
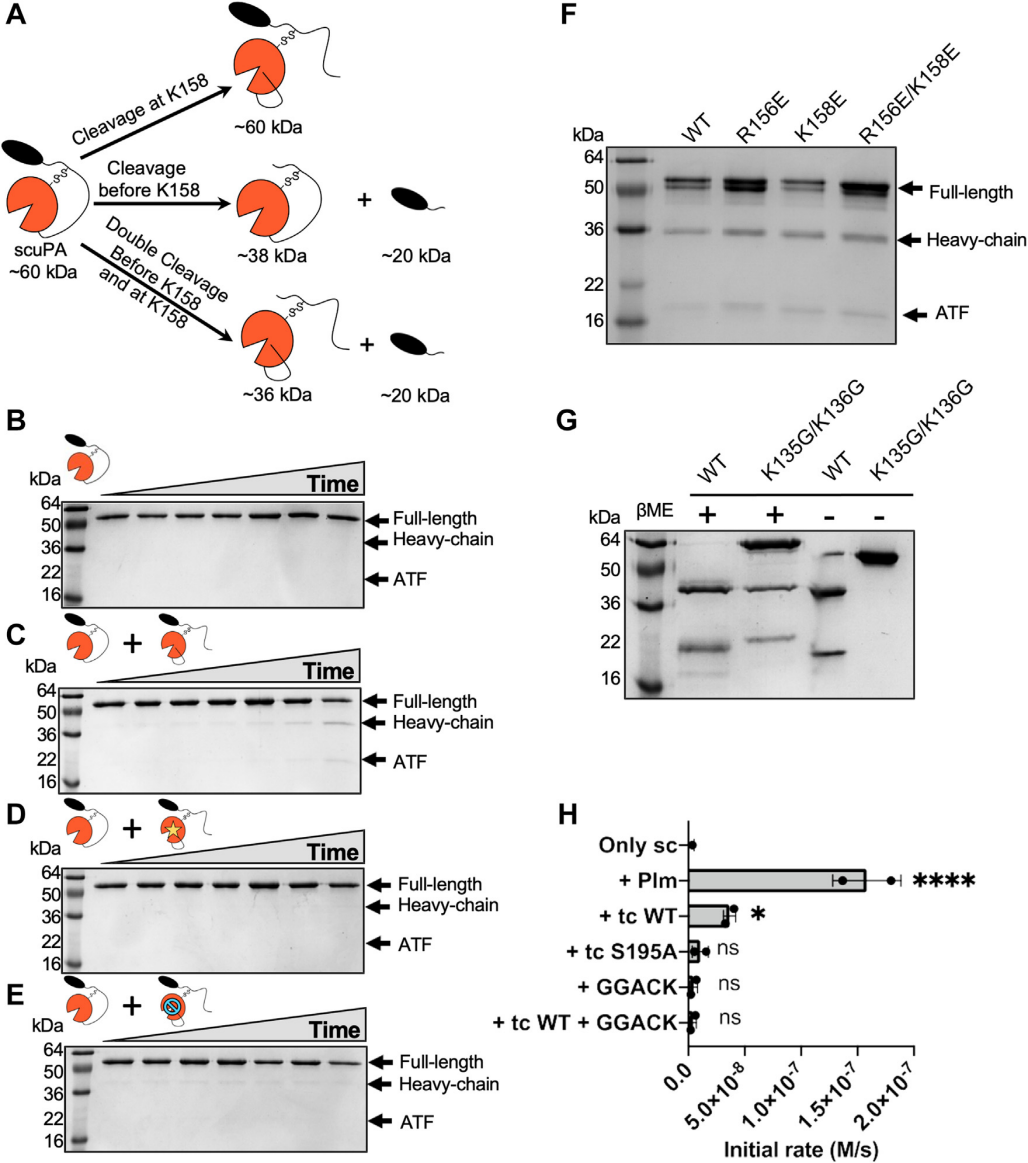
**TcuPA also cleaves scuPA between K135 and K136 in the linker before C148(1**_**CT**_**).***A*, scuPA S356(195_CT_)A (2 μM) was incubated at 37 °C in the absence of any other protease (*A*), in the presence of 0.2 μM tcuPA WT (*B*), in the presence of 0.2 μM tcuPA S356(195_CT_)A (*C*), or in the presence of GGACK-inhibited tcuPA WT (*D*). The reaction products were visualized by SDS-PAGE under nonreducing conditions. The reaction time points shown correspond to 0, 10 min, 30 min, 1 h, 3 h, 5 h, and 19 h. *E, cartoon model* showing where scuPA is cleaved in the linker to release the protease domain from the ATF. ScuPA can be cleaved yielding two polypeptide chains that can be separated under reducing conditions but remain disulfide bonded under nonreducing conditions. In a nonreducing SDS-PAGE, the higher molecular weight fragment (∼38 kDa) corresponds to the heavy chain of uPA, which contains the protease domain and part of the linker including C148(1_CT_). The lower molecular weight fragment corresponds to the light chain (ATF and the rest of the linker, ∼20 kDa). *F*, SDS-PAGE under nonreducing conditions showing the scuPA mutant proteins (WT, R156E, K158E, and R156E/K158E) after 30 days of storage at 4 °C. *G*, ScuPA WT and the double mutant K135G/K136G were incubated at 4 °C for more than 30 days. The autoactivation products were visualized by SDS-PAGE under reducing (+β-ME) and nonreducing conditions (−β-ME). *H*, scuPA K135G/K136G (2 μM) was incubated in the presence of plasmin (0.01 μM), tcuPA WT (0.2 μM), tcuPA S356(195_CT_)A (0.2 μM), GGACK (50 μM) or tcuPA WT (0.2 μM) previously inhibited with GGACK (50 μM) for 3 h at 37 °C. The amidolytic activity of the reaction products was assayed, and the basal activity of the added tcuPA was subtracted from the reactions where that enzyme was added. The bars represent the average of two technical replicates, which are shown as *dots* on each bar. βME, beta-mercaptoethanol; ATF, amino terminal fragment; GGACK, Glu–Gly–Arg–chloromethyl ketone; scuPA, single-chain uPA; tcuPA, two-chain uPA; uPA, urokinase-type plasminogen activator.

It has been reported that plasmin can also cleave in the linker between K135 and K136 to yield “LMW” uPA, which corresponds to the protease domain and a short stretch of the linker including C148(1_CT_) (13, 14, 15, 16). A cleavage between K135 and K136 would be consistent with the cleavage that yielded the ∼38 kDa band after storage of scuPA K158E and R156E (Fig. 6*E*). We hypothesized that tcuPA could cleave scuPA at this site and would effectively release the protease domain from the ATF, as the cleavage is upstream of the disulfide bond that tethers them together (Fig. 7*A*). To test this hypothesis, the catalytically dead mutant of scuPA S356(195_CT_)A (2 μM) was incubated in the presence or the absence of tcuPA WT or tcuPA S356(195_CT_)A or tcuPA WT inhibited by GGACK (Fig. 7, *B*–*E*). The reaction products were visualized by SDS-PAGE under nonreducing conditions, as the release of the protease domain would only be observed if there was some cleavage happening in the disordered liner before C148(1_CT_) (Fig. 7*A*). In agreement with our hypothesis, a ∼38 kDa was only observed when tcuPA WT was added, and no band was observed in any of the other conditions, suggesting that this cleavage depends solely on tcuPA WT. To check that this cleavage was independent of cleavage in K158 or R156, the autoactivation products of scuPA WT, R156E, K158E, and R156E/K158E after 30 days of storage at 4 °C were analyzed by SDS-PAGE under nonreducing conditions (Fig. 7*F*). All these proteins were cleaved before C148(1_CT_), yielding a band at ∼38 kDa. Isolation of these gel bands (from Fig. 7*F*) followed by tryptic digestion and MS/MS sequencing identified that the 38 kDa protein band corresponded to the protease domain with some part of the linker, and the 20 kDa protein band corresponded to the ATF (Fig. S8). We mutated both lysines to glycines and demonstrated that the K135G/K136G double mutant protein did not show a protein band corresponding to the heavy chain under nonreducing conditions indicating that cleavage must be occurring at one of these lysines (Fig. 7*G*). On the other hand, the SDS-PAGE under reducing conditions revealed that scuPA K135G/K136G mutant protein became cleaved at K158 consistent with activity assays of the single-chain K135/K136G protein incubated at 37 °C for 3 h, which showed that either plasmin or tcuPA could activate this protein (Fig. 7*H*). Our results demonstrate that tcuPA can cleave scuPA in the disordered linker at either K135 and/or K136, as well as after K158, but only this latter cleavage causes the protein to become active.

### Plasmin cleaves scuPA faster than scuPA but only after K158

**Figure 8:**
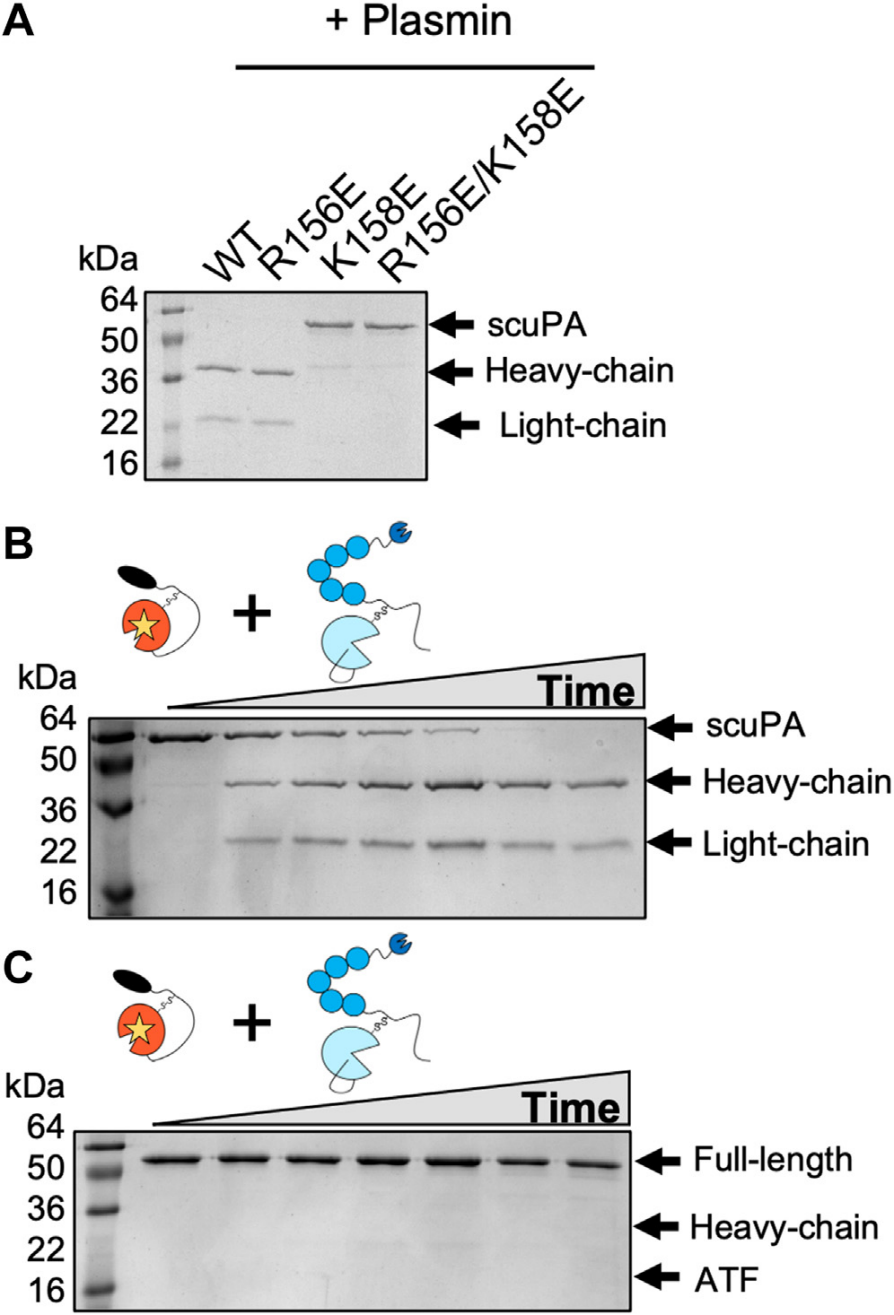
**Plasmin cleaves scuPA faster than tcuPA, and plasmin cleaves only after K158.***A*, the scuPA mutants (WT, R156E, K158E, and R156E/K158E, 1.5 μM) were incubated with plasmin (7.5 nM) at room temperature for 23 h, and the reaction products were visualized by SDS-PAGE under reducing conditions. *B* and *C*, scuPA WT (2 μM) was incubated at 37 °C in the presence of plasmin (0.01 μM) for 0, 10 min, 30 min, 1 h, 3 h, 5 h, and 19 h. The reaction products were visualized by SDS-PAGE under reducing (*B*) and nonreducing conditions (*C*). scuPA, single-chain uPA; tcuPA, two-chain uPA; uPA, urokinase-type plasminogen activator.

We also compared the efficiency of tcuPA-dependent activation of scuPA relative to plasmin-dependent activation. We examined plasmin cleavage of the scuPA mutants R156E, K158E, and R156E/K158E after incubating scuPA (1.5 μM) with plasmin (7.5 nM) after 24 h at 22 °C (Fig. 8*A*). Whereas we showed that tcuPA could cleave the K158E mutant at position 158, plasmin was not able to cleave either the K158E or the R156E/K158E mutants, suggesting that plasmin is a more specific activator of scuPA than tcuPA and that it only cleaves at K158. To study the kinetics of plasmin-dependent activation of scuPA, scuPA S356(195_CT_)A (2 μM) was incubated in the presence of plasmin (10 nM) at 37 °C and the reaction progression was monitored by SDS-PAGE under reducing and nonreducing conditions (Fig. 8, *B* and *C*). Plasmin was able to cleave scuPA WT after K158 1000-fold faster than tcuPA WT under the same reaction conditions (Figs. 5*B* and 8B). Plasmin was also incapable of cleaving scuPA between K135/K136 as observed in the SDS-PAGE under nonreducing conditions (Fig. 8*C*). These results demonstrate that plasmin is a more efficient activator of scuPA than tcuPA; however, tcuPA can activate scuPA and can also release the protease from its uPAR-bound form whereas plasmin cannot.

## Discussion

In the work presented here, we set out to characterize the dynamics and activity of scuPA and tcuPA. The HDX-MS results show that tcuPA is generally less dynamic than scuPA. Decreased dynamics in the loops of the C-terminal β-barrel would cause the substrate-binding site to become more ordered, likely improving substrate binding in tcuPA. The ordering of β9-strand and the 170s loop in tcuPA would correspond to increased catalytic efficiency and was also observed in murine uPA in the presence of an activating antibody (25, 26). A crystal structure of murine uPA shows that this protein could adopt a non–chymotrypsin-like fold in which the β9-strand is flipped by 180° and the catalytic serine located downstream of the 180s loop was not correctly positioned for catalysis (26). Therefore, the positioning and dynamics of the β9-strand can be considered an important indicator for catalysis. In addition, previous NMR studies showed that the β-strand connecting the 90s loop with the 70s loop in the N-terminal β-barrel of thrombin has a role in mediating motions between the 90s and the 70s loops (45). These motions were not visualized by HDX-MS and were only evident by NMR. We cannot discard the idea of the β9-strand in uPA could also be mediating the allostery between the 170s and 180s loops.

It is interesting that HDX-MS studies of various coagulation proteases show that activation has varying effects. The dynamics of the protease domains of factor IX and factor VII do not change upon activation (21, 22). This is consistent with these proteases not being active after cleavage and requiring the binding of a cofactor to become fully active. Factor XI also requires a cofactor to become fully active; however, activation of factor XI causes decreased dynamics in the 140s loop and the dynamics of the sequence connecting the extra domains located in the light-chain N terminal to the protease domain (23). Comparison of thrombin to its single-chain prethrombin-2 form also showed decrease in dynamics in the 140s loop upon activation; however, thrombin also has increased dynamics in the 70s and 110s loops comprising the anion binding exosite I, the 90s loop, and the C-terminal helix (24).

Our results demonstrate that freshly purified scuPA has no activity above the limit of detection of the amidolytic assays presented here. However, storage even at 4 °C results in increased amidolytic activity suggesting that tcuPA can be generated over time and is able to cleave scuPA *in vitro*. Importantly, only the cleavage between K158(15_CT_) and I159(16_CT_) produces active uPA. The mechanism by which the first tcuPA is generated remains an open question; however, the S356(195_CT_)A mutant scuPA did not become cleaved upon storage strongly suggesting that the activation of scuPA during storage is due to the production of tcuPA from scuPA and not from some extraneous protease in the protein preparation. The most likely possibility is that a molecule of scuPA could stochastically acquire a conformation where the catalytic triad is ready for catalysis. After the first molecule of tcuPA is generated, tcuPA-dependent activation of scuPA then occurs and leads to the accumulation of active tcuPA. The fact that uPA can autoactivate explains the controversy in the literature about whether scuPA is active or not toward its substrates S2444 and plasminogen, since activity would depend on preparation and storage time in an unpredictable manner (10, 11, 12).

We also observed autoactivation of the so-called “uncleavable” scuPA mutant K158E. The K158E mutant was previously used by other authors who assumed it would never become active because plasmin cannot cleave if K158 is mutated (12, 46, 47, 48, 49, 50). We confirmed that these mutants could not be activated by plasmin, but we found that tcuPA could cleave scuPA at position 158 even when this site was mutated to a glutamate. We observed that as with WT uPA, freshly purified K158 mutants did not have any measurable activity, but their activity increased over time of storage. We also demonstrated that once the proteins (whether mutant or WT) were cleaved at position 158, they became active and were able to promote autoactivation as well as tcuPA WT. Previous authors had mutated K158 to Gly, Glu, Thr, Val, and Met and found that these mutants did have some activity (51, 52, 53, 54, 55). It is important to note that the new N terminus, which is required for ordering the catalytic triad, would be the same regardless of the side chain occupying position 158. Interestingly, some of the previous articles show the SDS-PAGE of their “single-chain” mutant protein, and it is very evident that there is a band at ∼36 kDa that could correspond to some version of the heavy chain of uPA (53, 54) suggesting that the observed “intrinsic activity” was most likely because of the cleavage of even these mutants at position 158.

Our results show that plasmin and tcuPA can cleave scuPA. While plasmin can only cleave after K158, tcuPA can cleave after K135/K136 and K158, even when this last position is mutated. Previous authors reported (15) that plasmin was also able to cleave at K135 after incubating scuPA WT in the presence of plasmin and observing cleavage at that position. Our results demonstrate that it must have been autoactivated tcuPA present in their sample that cleaved after K135, not plasmin.

Previous authors have also shown that chicken uPA can autoactivate much faster than human uPA (34, 35). These two proteins only share 30% sequence identity. The linker in the light chain is longer in human uPA. On the contrary, the 60s loop and the sequence after the C-terminal helix and the N-terminal sequence before the EGF-like domain are longer in chicken uPA. By replacing the first ten residues of human uPA with the first 20 residues of chicken uPA, Aimes *et al.* (34) reported that the autoactivation of human uPA was accelerated to the same degree of chicken uPA. It remains unknown how the dynamics of both proteins differ, regardless of the sequence identity and the loop length differences. In addition, birds do not have a gene homologous to the uPAR (56), suggesting that uPA may serve a different role in birds than in mammals.

**Figure 9:**
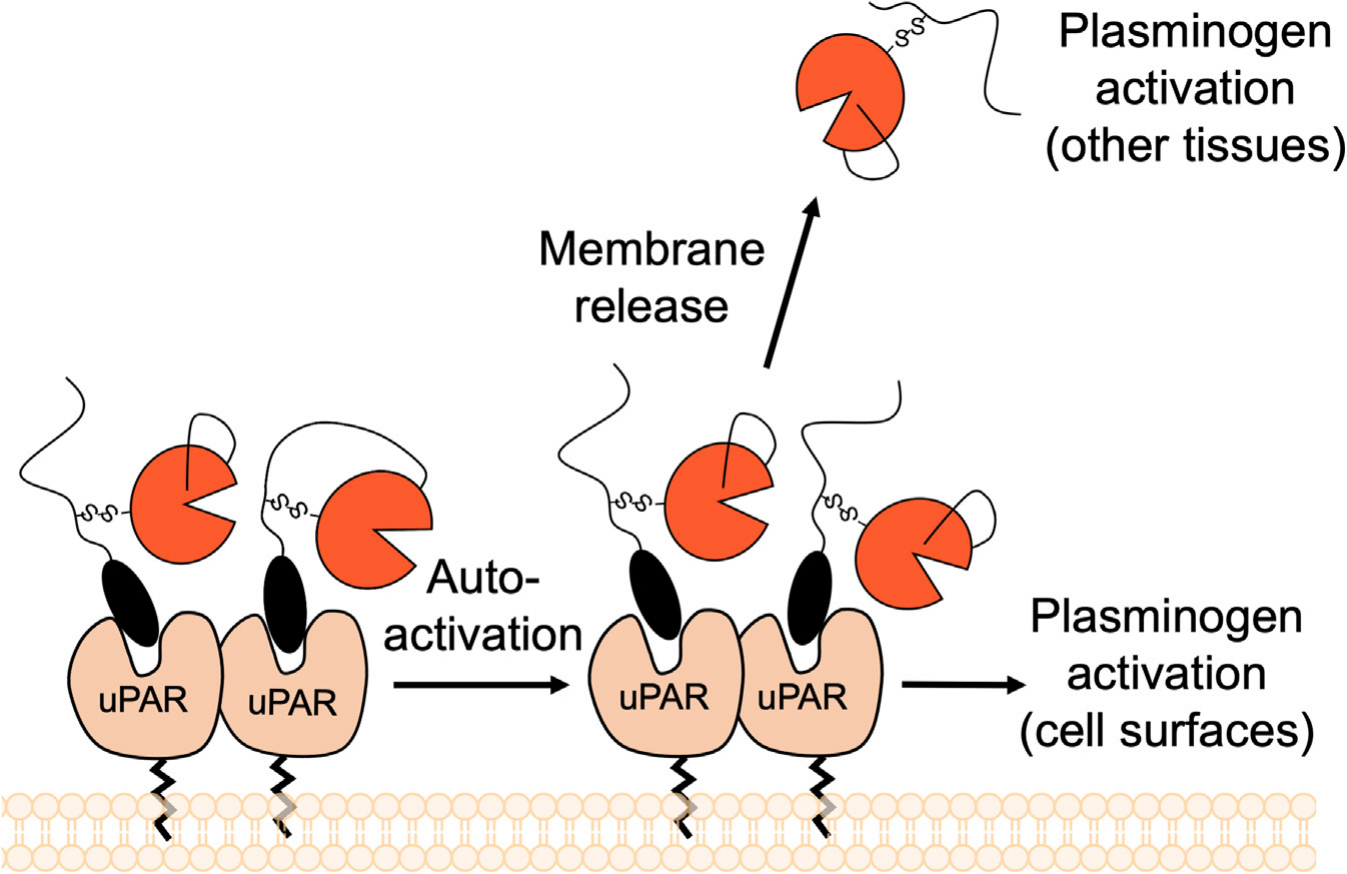
**Schematic model of scuPA autoactivation.** On cell surfaces, scuPA and tcuPA are localized to the cell membrane by interaction with the uPAR. The uPAR can exist in monomeric or dimeric forms, both of which bind uPA with the same affinity (7). The dimerization of uPAR brings two molecules of uPA together and promotes mutual activation. The activation of scuPA into tcuPA in the cell surface enables activation of plasminogen molecules attached to the membrane. In addition, uPA could also catalyze a cleavage in the linker that would release active tcuPA from the membrane and promote plasminogen activation on other tissues. scuPA, single-chain uPA; tcuPA, two-chain uPA; uPA, urokinase-type plasminogen activator; uPAR, uPA receptor.

Previous authors have reported that scuPA can be converted into tcuPA on the cell surface by a plasmin-independent mechanism (57), suggesting that autoactivation could happen *in vivo*. The uPA is localized at the cell surface by binding to uPAR, a membrane-attached protein that has been reported to dimerize (6, 7). Based on our results, we predict that the uPAR dimers could bring two molecules of uPA close together and could promote autoactivation on the cell surface (Fig. 9). The proximity of two molecules of uPA bound to a uPAR dimer could result in autoactivation of scuPA in a manner similar to what we observed upon storage. The small amount of tcuPA generated by autoactivation could then start the plasmin–uPA positive feedback loop on cell surfaces. Cell surface plasmin might also come into close contact with scuPA and efficiently cleave it to active tcuPA. It remains unclear how much of one mechanism *versus* the other is operating under different cellular conditions. We also demonstrated that tcuPA can cleave scuPA after K135/K136 in the linker region, thus releasing the “LMW” form of uPA into solution. The soluble uPA generated by this pathway could be the causative agent for further protease activation in such processes as metastasis.

## Experimental procedures

### Expression of full-length human uPA

The gene for full-length human uPA was synthesized with codons optimized for expression in *E. coli* by Genewiz. The protein was designed to have a C-terminal 6× His tag and tobacco etch virus protease cleavage site after the uPA sequence. The uPA gene was cloned into an ampicillin-resistant plasmid (pFLhuPA) containing a T7 promoter, which is the same backbone previously used for expression of murine uPA (26).

To express uPA, *E. coli* BL21(DE3) cells were freshly transformed with pFLhuPA. A 10 ml overnight starter culture was grown from a single colony in M9ZB media containing 200 mg/l ampicillin (M9ZB-Amp). The starter culture was transferred to 1 l of M9ZB-Amp media and grown at 37 °C to an absorbance of 0.6 at 600 nm. The cells were induced with 1 mM of IPTG for 4 h at 37 °C and then harvested by centrifugation at 5000*g* for 15 min at 4 °C. The cell pellets were stored at −80 °C until use.

### Refolding of full-length uPA

A frozen cell pellet from 1 l of culture was thawed and resuspended in sonication buffer (50 mM Tris [pH 8], 0.5 M NaCl, 1 mM EDTA, 10% glycerol, 1 mM beta-mercaptoethanol [β-ME], and 1% Triton X-100 [v/v]). The cells were lysed by ice-cold sonication using four intercalated pulses of 45 s with 2 min rest in between. The sonication lysate was treated with 300 μg of DNAse I (Roche Diagnostics GmbH), 6 mM MnCl_2_, and 6 mM MgCl_2_ for 45 min at 25 °C with rocking. The cell lysate was centrifuged at 17,000*g* for 15 min at 4 °C, and the supernatant was discarded. The pellet that contained the inclusion bodies was washed with sonication buffer three times with decreasing concentrations of Triton X-100 (1%, 0.25%, and 0%, respectively). The inclusion bodies were collected by centrifugation at 17,000*g* for 10 min at 4 °C between washes. The inclusion bodies were solubilized in denaturing buffer (50 mM Tris [pH 8], 100 mM NaCl, 6 M urea, 1 mM EDTA, and 10% glycerol) containing 10 mM β-ME at 4 °C for 4 h. The solubilized inclusion bodies were centrifuged at 17,000*g* for 15 min at 4 °C. A portion (30–75 mg) of the solubilized protein was diluted to 0.15 mg/ml in denaturing buffer with 1 mM β-ME, placed in dialysis tubing 12 to 14 kDa cutoff, and dialyzed against 4 l of dialysis buffer (50 mM Tris [pH 8], 10% glycerol, and 150 mM NaCl) containing 3 M urea and 1 mM β-ME for 8 to 12 h at 4 °C. The buffer was changed two more times to 4 l of dialysis buffer (50 mM Tris [pH 8], 10% glycerol, and 150 mM NaCl) for 8 to 12 h each at 4 °C. After dialysis, the soluble refolded protein was centrifuged at 22,000*g* for 15 min at 4 °C and subsequently filtered using a 0.22 μm pore filter to eliminate aggregated proteins.

### Purification of full-length uPA

The refolded protein was purified on His Pur nickel–nitrilotriacetic acid resin (Thermo Fisher Scientific). The resin was pre-equilibrated in PBS (10 mM Na_2_HPO_4_, 1.8 mM KH_2_PO_4_, 2.7 mM KCl, 137 mM NaCl, pH 7.4) with 25 mM imidazole, pH 7.4. The refolded soluble protein solution was brought to 25 mM of imidazole and loaded onto the column at 4 °C. The resin was washed with four column volumes (CVs) of PBS with 25 mM imidazole, 4 CVs of PBS with 25 mM imidazole containing an additional 150 mM NaCl, and finally with 4 CVs of PBS with 25 mM imidazole. The protein was eluted in 5 ml fractions with 30 ml PBS containing 400 mM imidazole into tubes containing 5 ml of PBS. The fractions containing uPA were dialyzed against PBS at 4 °C overnight.

To obtain the scuPA, the purified protein after nickel–nitrilotriacetic acid purification was stored in 10 mM benzamidine to avoid self-cleavage. Right before usage, the protein was finally purified on a HiLoad 16/60 Superdex 75 prep grade column (GE Healthcare) in PBS at 22 °C.

To obtain the two-chain version of uPA, the protein was cleaved with plasmin (Haematologic Technologies) during the final dialysis step in PBS at a 1:200 ratio of plasmin:uPA at 4 °C for 16 h. Cleavage was monitored by SDS-PAGE. The reaction was stopped by adding 0.1 μM aprotinin (Millipore Sigma) and 10 mM benzamidine. tcuPA was purified by size exclusion on a Superdex 75 column.

Site-directed mutagenesis (Dpn I protocol) was used to generate the uPA mutants. All mutants were purified using the same protocol described previously.

Protein concentration was measured by the absorbance at 280 nm, using a Nanodrop. The extinction coefficient at 280 nm of uPA was 73,810 M^−1^ cm^−1^, when all cysteines were oxidized, and 72,310 M^−1^ cm^−1^ when all cysteines were reduced.

### Amidolytic activity assay

The amidolytic activity of uPA was measured at 10 nM of uPA in 200 μl in PBS (pH 7.4) in a 96-well microplate using the chromogenic substrate analog pyro–Glu–Gly–Arg–pNA or DPG444 (DiaPharma) (also referred to as S2444). Substrate concentrations ranged from 10 μM to 500 μM. The reaction components were incubated for 10 min at 37 °C before adding uPA (preincubated at 37 °C) to start the reaction. The reaction was monitored for 10 min at 37 °C, and the absorbance at 405 nm was measured every 20 s. Initial rates were calculated using data from only the first 2 min. Reactions were run in duplicate.

### Plasmin generation activity assay

The ability of uPA to activate plasminogen was measured at 10 nM of uPA in PBS (pH 7.4) using human Glu-Plasminogen (Haematologic Technologies) and 0.5 mM of the plasmin-specific chromogenic substrate analog H–D–Val–Leu–Lys–pNA or S2251 (DiaPharma). The Glu–plasminogen concentration was varied from 0.5 μM to 10 μM. All the reaction components were preincubated at 37 °C for 10 min prior to mixing. The reaction was started by the addition of uPA, and the absorbance at 405 nm was monitored every 20 s for 10 min at 37 °C. The initial rates were calculated using the approach described (58) after determining the *K*_*M*_ and *k*_cat_ of plasmin for S2251 under our reaction conditions (*K*_*M*_ = 0.2 mM and *k*_cat_ = 13.7 s^−1^):

$$\text{Absorbance}\;\text{at}\;405\;\text{nm}\;=\;\left(2.63\;\times \;10^{4}\frac{M}{s}\right)\frac{V_{\max}\left[\text{Plasminogen}\right]}{K_{M}\;+\;\left[\text{Plasminogen}\right]}t^{2}$$

The initial rates were obtained by plotting the absorbance at 405 nm divided by the conversion factor in brackets against the time squared. Initial rates were calculated from the first 3 min of the reaction.

### HDX-MS

HDX-MS was performed using a Waters Synapt G2Si system with HDX technology (Waters Corporation) (59, 60, 61). The H_2_O buffer was PBS, which was lyophilized and resuspended in deuterium oxide (D_2_O) to prepare the D_2_O buffer. A 4 μl portion of a 10 μM protein sample was incubated for 5 min at 22 °C and then mixed with 56 μl of water buffer as a control or D_2_O buffer for deuteration times of 0, 30, 60, 120, and 600 s. We previously showed that measurement of amide exchange during this time regime best captures allosteric transitions (39, 40). The reaction was quenched with 60 μl of 250 mM Tris(2-carboxyethyl)phosphine, pH 2.5, at 0 °C. A portion of the quenched sample (50 μl) was injected into a sample loop and subsequently digested on an in-line pepsin column (Immobilized Pepsin; Pierce, Inc) at 15 °C. The resulting peptides were captured on a BEH C18 Vanguard precolumn, separated by analytical chromatography (Acquity UPLC BEH C18, 1.7 μM, 1.0 × 50 mm; Waters Corporation) using a 7 to 85% acetonitrile in 0.1% formic acid over 7.5 min at a flow rate of 40 μl/min, and electrosprayed into the Waters Synapt G2Si quadrupole time-of-flight mass spectrometer. The mass spectrometer was set to collect data in the mobility, electrospray ionization in positive mode; mass acquisition ranges from 200 to 2000 (*m/z*); and scan time of 0.4 s. Continuous lock mass correction was accomplished with infusion of leu-enkephalin every 30 s (mass accuracy of 1 ppm for calibration standard). For peptide identification, the mass spectrometer was set to collect data in MS^E^, mobility electrospray ionization in positive mode instead. Peptide masses were identified from triplicate analyses of 10 μM protein, and data were analyzed using PLGS 2.5 (Waters Corporation). Peptide masses were identified using a minimum number of 250 ion counts for low-energy peptides and 50 ion counts for their fragment ions.

The peptides identified in PLGS were then analyzed in DynamX 3.0 (Waters Corporation) using a cutoff score of 6.0, error tolerance of 5 ppm, and requiring that the peptide be present in at least two of the three identification runs. The relative deuterium uptake for each peptide was calculated by comparing the centroids of the mass envelopes of the deuterated samples *versus* the undeuterated controls (59). For all HDX-MS data, at least two biological replicates were analyzed each with three technical replicates. Data are represented as mean values ± SEM of three technical replicates because of processing software limitations; however, the LEAP robot provides highly reproducible data for biological replicates. The deuterium uptake was corrected for back-exchange using a global back exchange correction factor (typically 25%) determined from the average percent exchange measured in disordered regions (62). ANOVA analyses and *t* tests with a *p* value cutoff of 0.05 implemented in the program, DECA, were used to determine the significance of differences between HDX data points (62). Deuterium uptake plots were generated in DECA (github.com/komiveslab/DECA), and the data are fitted with an exponential curve for ease of viewing.

### The cleavage of scuPA during storage in the absence of external proteases

After removing the benzamidine by size-exclusion chromatography, the uPA proteins were concentrated using a Vivaspin 6 molecular weight cutoff of 10 kDa concentrator until the absorbance at 280 nm was equal or greater than 0.1. The proteins were immediately assayed to provide the “day 1” measurement and then stored at 4 °C for 30 days and assayed again. SDS-PAGE under reducing conditions was used to visualize the cleavage of the proteins.

The amidolytic activity of the proteins on “day 1” and “day 30” was measured by incubating 100 nM of scuPA in 200 μl in PBS (pH 7.4) in a 96-well microplate using 50 μM of S2444 as a substrate. The reaction was started by the addition of the enzyme, and the absorbance at 405 nm was measured every 20 s for 5 min. All the reagents were preincubated at 37 °C for 10 min before the reaction. Each reaction was run in duplicate.

In the case of the autoactivation of scuPA S356(195_CT_)A, the single-chain form of this mutant protein and of scuPA WT, which were both stored individually with 10 mM benzamidine as previously, was concentrated to 2 ml, and purified by size-exclusion chromatography as described previously. This modification allowed for the proteins to elute from the column at 3 μM, and no further concentration was needed. The single-chain proteins without benzamidine were stored at 4 °C for 10 days, and cleavage was evaluated by SDS-PAGE under reducing conditions.

Storage of scuPA K135G/K136G was carried out with the same protocol as the WT protein.

### The tcuPA-dependent cleavage of scuPA

To test whether tcuPA WT could cleave scuPA, 2 μM of scuPA WT or S356(195_CT_)A were incubated in PBS in the absence of any protease or in the presence of 10 nM plasmin (1:200 ratio), 200 nM tcuPA WT (1:10 ratio), 200 nM tcuPA S356(195_CT_)A, or 200 nM GGACK-inhibited tcuPA WT. A reaction with only 200 nM tcuPA WT was also carried out in the absence of scuPA to account for any activity of that additional enzyme. The reaction was measured after 0, 10 min, 30 min, 1 h, 3 h, 5 h, and 19 h at 37 °C. The progress of the reaction was monitored by measuring the amidolytic activity of the reaction products or SDS-PAGE under reducing and nonreducing conditions. The amidolytic activity of the proteins was quantified by reacting 100 nM of the reacted scuPA with 100 μM of S2444 in PBS (pH 7.4) at 37 °C. To obtain the GGACK-inhibited plasmin-treated tcuPA WT, 2 μM of tcuPA WT was incubated with 50 μM GGACK at 22 °C for 30 min.

### Amidolytic activity of scuPA K135G/K136G

To test whether scuPA K135G/K136G could become activated by tcuPA WT or by plasmin, 2 μM of scuPA K135G/K136G were incubated in PBS in the absence of any protease or in the presence of 10 nM plasmin (1:200 ratio), 200 nM tcuPA WT (1:10 ratio), 200 nM tcuPA S356(195_CT_)A, or 200 nM GGACK-inhibited tcuPA WT. A reaction with only 200 nM tcuPA WT was also carried out in the absence of scuPA to account for any activity of that additional enzyme. To obtain the GGACK-inhibited plasmin-treated tcuPA WT, 2 μM of tcuPA WT was incubated with 50 μM GGACK at 22 °C for 30 min before the incubation with scuPA K135G/K136G, as explained previously. The cleavage of scuPA K135G/K136G was carried out for 3 h at 37 °C. Activation was quantified by measuring the amidolytic activity of the proteins by reacting 100 nM of the reacted scuPA with 100 μM of S2444 in PBS (pH 7.4) at 37 °C for 5 min. The activity of the control tcuPA WT was subtracted to correct for basal tcuPA activity. Statistical significance was quantified by unpaired *t* test.

### Plasmin-dependent activation of scuPA mutants

Plasmin-dependent and the tcuPA WT–dependent activation of scuPA WT or mutants was measured by incubating 1.5 μM of scuPA with 7.5 nM of plasmin or tcuPA WT in PBS (pH 7.4) for 24 h at room temperature. The reaction products were assayed by SDS-PAGE under reducing conditions.

### Sequencing of uPA using MS

The autoactivation products of scuPA, 1.5 μM of scuPA, were stored for 30 days at 4 °C at a concentration of 1 to 3 μM. The samples were analyzed by 15% SDS-PAGE at 150 V for 70 min. The gel was stained for 15 min in 0.25% Coomassie Blue G-250, 40% methanol, 10% acetic acid and destained overnight in 20% methanol and 10% acetic acid. The selected protein gel bands were excised, cut into small pieces, and washed as described previously (63, 64). The cysteines of proteins in the gel bands were reduced and alkylated with iodoacetamide, prior to trypsin digestion. The proteins were trypsinized for 45 min at 4 °C and incubated at 37 °C overnight. The supernatant containing the tryptic peptides was dried in a speed vac and stored at −20 °C for further analysis.

Trypsin-digested samples were analyzed by LC–MS/MS using nanospray ionization on an Orbitrap fusion Lumos hybrid mass spectrometer (Thermo) interfaced with nanoscale reversed-phase UPLC (Thermo Dionex UltiMate 3000 RSLC Nano System) using a 25 cm, 75-micron ID glass capillary packed with 1.7 μm C18 (130) BEH beads (Waters Corporation) and separated on a linear gradient (5–80%) of acetonitrile at a flow rate of 375 nl/min for 1 h in 0.1% formic acid. Mass spectrometer parameters are as follows: (1) an MS1 survey scan using the orbitrap detector (mass range [*m/z*]: 400 to 1500 [using quadrupole isolation], 60,000 resolution setting, spray voltage of 2200 V, ion transfer tube temperature of 285 °C, automatic gain control target of 400,000, and maximum injection time of 50 ms). (2) Data-dependent scans (top speed for most intense ions, with charge state set to only include +2 to five ions, and 5 s exclusion time, while selecting ions with minimal intensities of 50,000 at which the collision event was carried out in the high-energy collision cell (higher energy collisional dissociation with a collision energy of 30%), and the fragment masses were analyzed in the ion trap mass analyzer (with ion trap scan rate of turbo, first mass *m/z* was 100, automatic gain control target of 5000, and maximum injection time of 35 ms). Protein identification and label-free quantification were carried out using Peaks Studio 8.5 (Bioinformatics Solutions, Inc).

## Data availability

The HDX-MS and in-gel trypsin digest data are available at massive.ucsd.edu under the dataset MSV000090611. We uploaded the HDX-MS raw data and the State Data Excel file that contains the information to make the uptake plots reported here.

## Supporting information

This article contains supporting information.

## Conflict of interest

The authors declare that they have no conflicts of interest with the contents of this article.
