# Supplementary material for: The autoactivation of human single-chain urokinase-type plasminogen activator (uPA)
Source: J Biol Chem. 2023 Aug 20;299(10):105179. doi: 10.1016/j.jbc.2023.105179 (PMC10520878; doi:10.1016/j.jbc.2023.105179)
Supplement: Supporting Figures S1–S8 and Table S1 [file mmc1.pdf]

The autoactivation of human single-chain urokinase-type plasminogen activator (uPA) can trigger plasmin-dependent fibrinolysis

Constanza Torres-Paris, Yueyi Chen, Lufan Xiao, Harriet J. Song, Pingyu Chen, Elizabeth A. Komives

SUPPORTING INFORMATION

Includes Supporting Figures S1 to S8 and Supporting Table S1.

SUPPORTING FIGURES

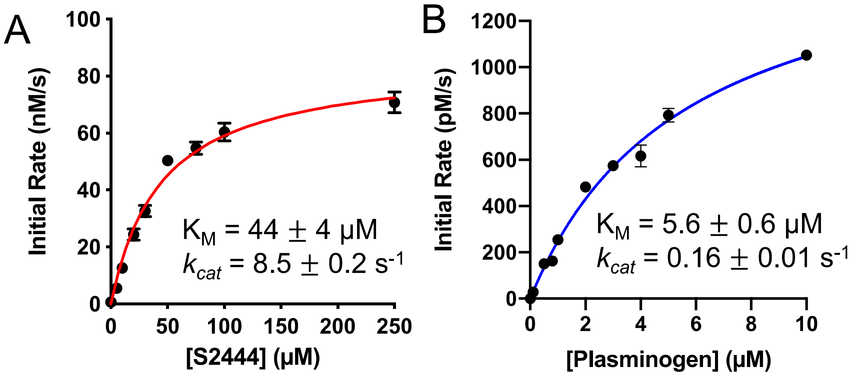

Figure S1. Activity of refolded uPA. (A) Michaelis-Menten plot of tcuPA (10 nM) towards the chromogenic substrate analog S2444 (5 μM – 1 mM). (B) Michaelis-Menten plot of tcPA (10 nM) towards Glu-plasminogen (0.1 - 10 μM). The plasmin generated was detected by the plasmin-specific chromogenic substrate S2251.

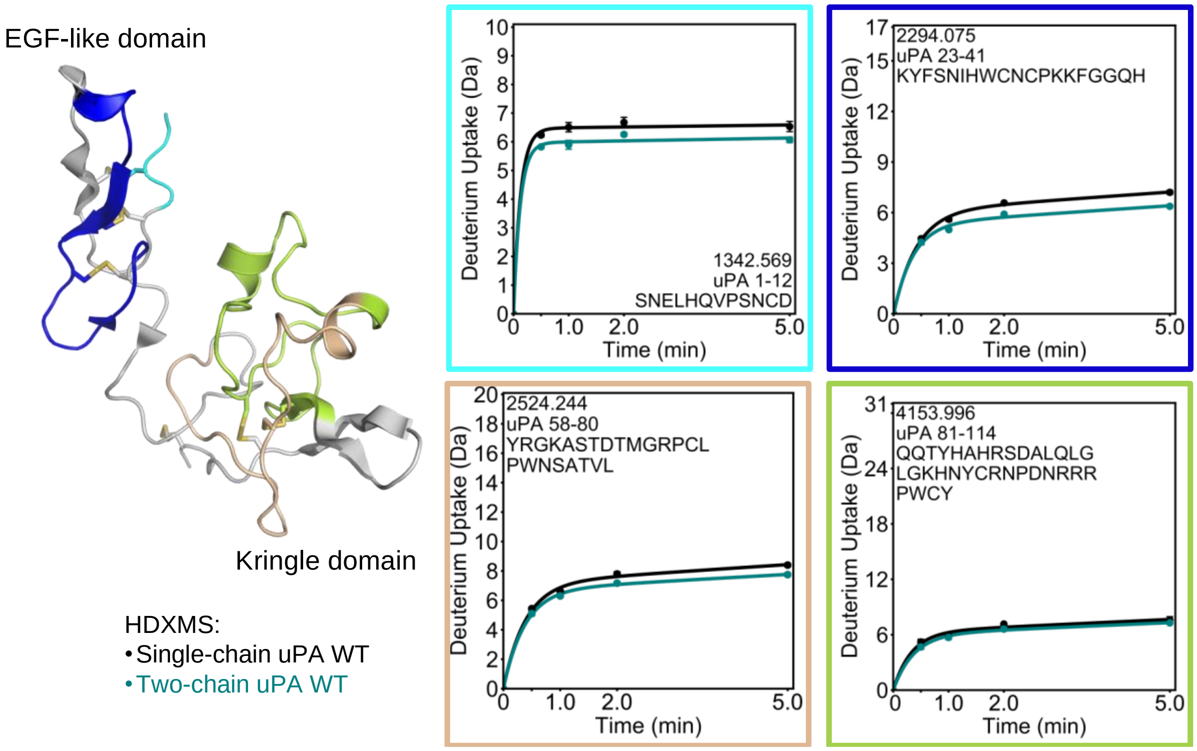

Figure S2. The dynamics of the ATF are not affected by plasmin cleavage of uPA. HDXMS revealed the dynamics of tcuPA WT (teal) and scuPA WT (black). The deuterium uptake plots for four representative peptides in the ATF are shown mapped on its crystal structure (PDB: 3BT2). The peptide spanning residues 1-12 is colored in cyan; the one spanning residues 23-41 in blue; the one spanning residues 58-80 in tan; and the one spanning residues 81-114 in green. The uptake plots are bordered in the same color scheme as on the structure. The side chains of the cysteines forming disulfide bonds are shown as sticks on the structure.

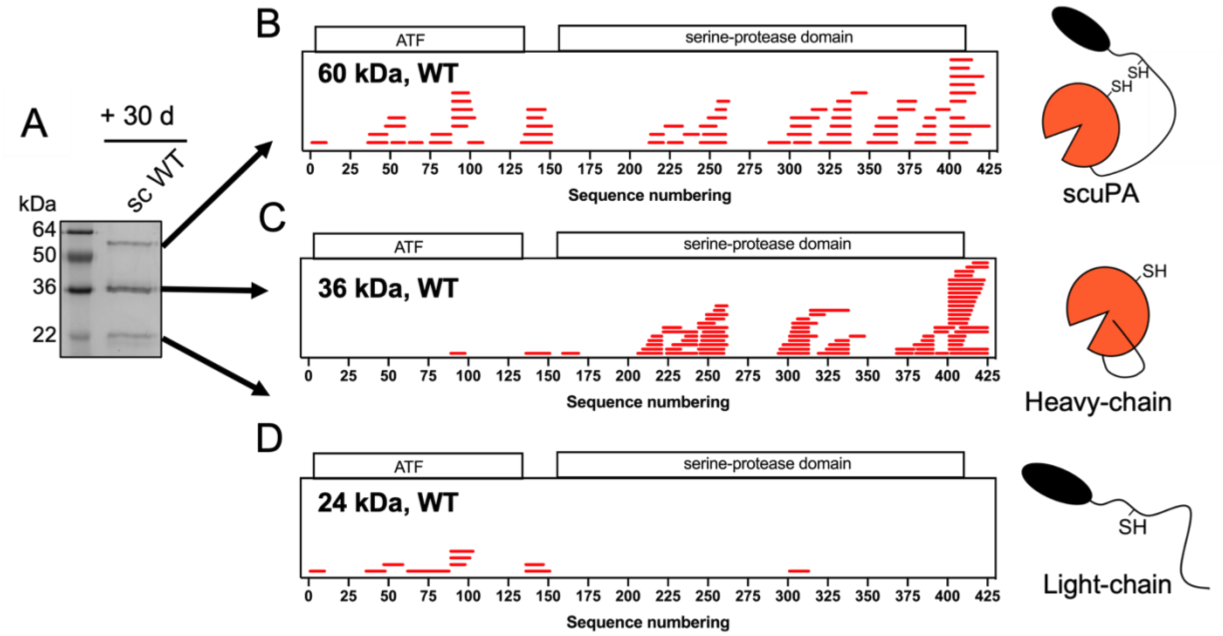

Figure S3. Coverage maps of the trypsin-digested MS/MS sequencing of the bands of scuPA WT after 30 days of storage at 4˚C.(A) SDS-PAGE under reducing conditions of the autoactivation products of scuPA WT. The bands of this gel were cut, trypsin-digested and sequenced by MS/MS. The peptides detected by MS are shown in the coverage maps as red lines over the sequence they cover. This gel is the same as in Fig 4B, right. (B) Coverage map of the 60 kDa band that constitutes the full-length scuPA. (C) Coverage map of the 36 kDa band that constitutes the heavy-chain of uPA. (D) Coverage map of the 24 kDa band that corresponds to the ATF and the linker.

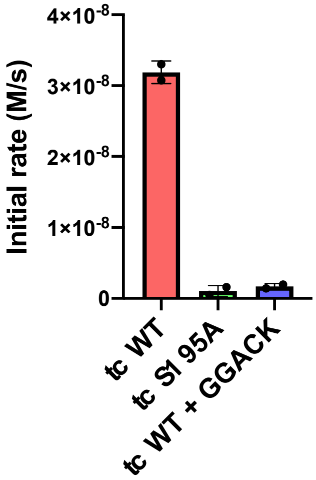

Figure S4. TcuPA S356(195A_{CT}) and tcuPA pre-incubated with the uPA-specific inhibitor GGACK do not have amidolytic activity. The amidolytic activity of tcuPA WT, tcuPA S356(195A_{CT}) or tcuPA pre-incubated with 50 µM of GGACK was measured by incubating 10 nM of the enzymes with 50 µM of S2444 for 37 ˚C. The reaction was monitored by measuring A_{405nm} every 20 s for 5 min. Each bar graph shows the average of two technical replicates, shown as individual data points on the bar graph, and the error bars represent the standard deviation of both replicates.

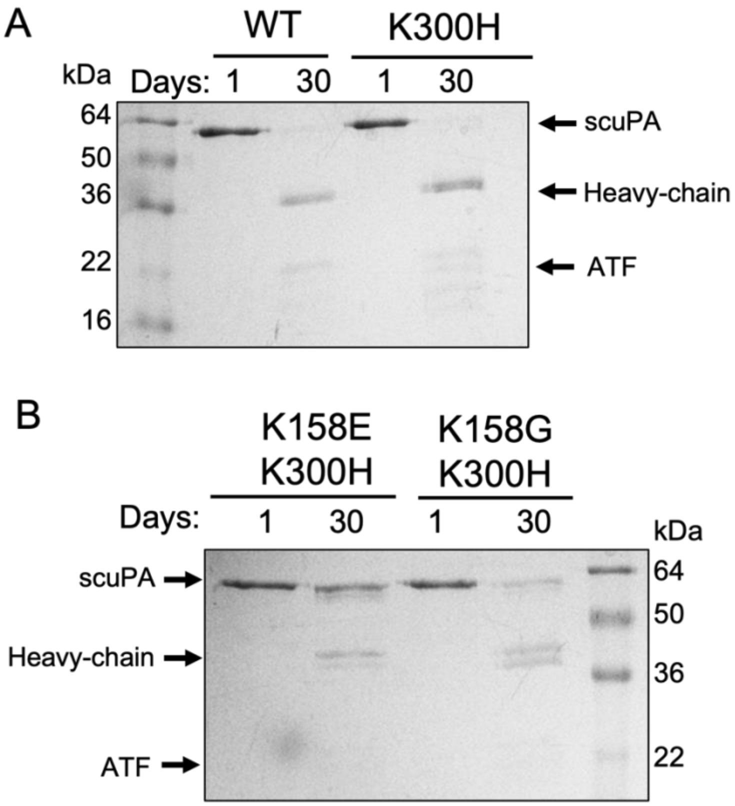

Figure S5. The mutation K300H in scuPA does not prevent autoactivation. (A) SDS-PAGE under reducing conditions showing sc uPA WT and K300H on the day of purification and after 30 days of storage at 4˚C. (B) SDS-PAGE under reducing conditions showing the double mutants sc uPA K158E/K300H and K158G/K300H on the day of purification and after 30 days of storage at 4˚C.

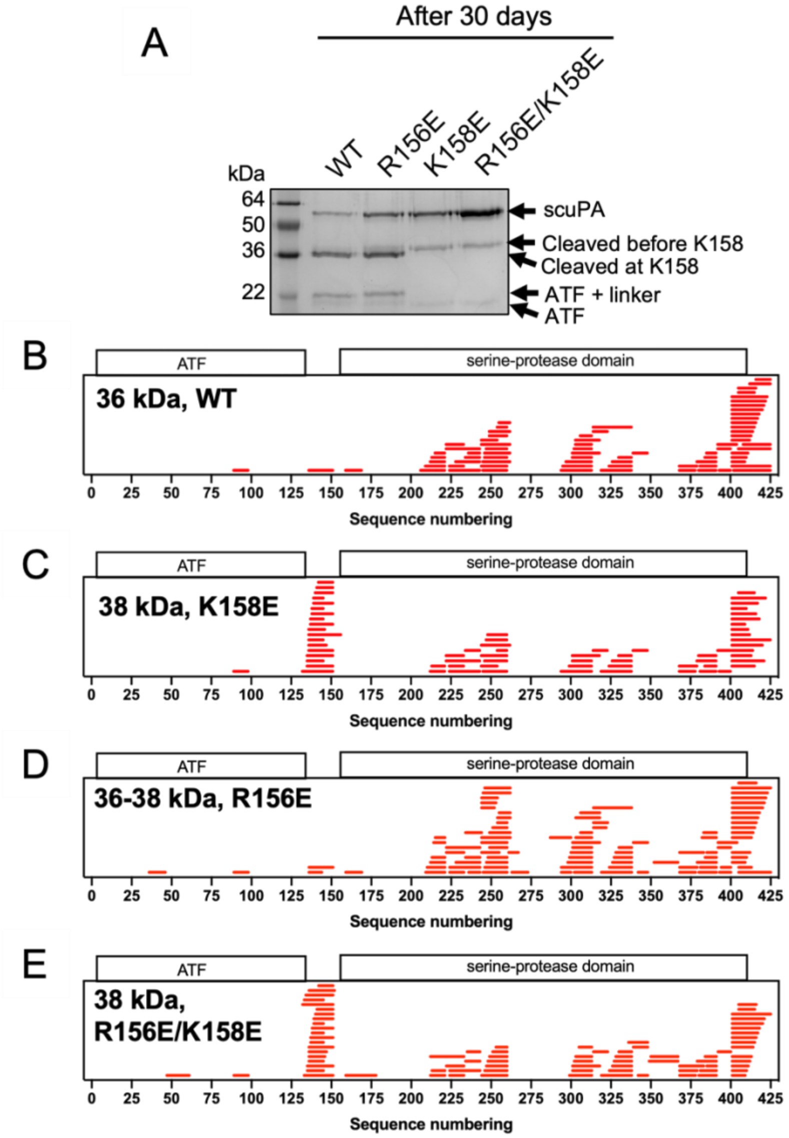

Figure S6. The 36 and 38 kDa bands product of the incubation of scuPA (WT, R156E, K158E and R156E/K158E) at 4˚C for 30 days corresponds to the heavy chain of uPA. (A) SDS-PAGE under reducing conditions showing the scuPA proteins (WT, R156E, K158E and R156E/K158E) after 30 days of storage at 4˚C. This gel is the same as in Fig. 6E. The bands of this gel were cut, trypsin-digested and sequenced by MS/MS. The peptides detected by MS are shown in the coverage maps as red lines over the sequence they cover. (B) Coverage map of the 36 kDa band of scuPA WT, which corresponds to the protease domain of uPA. (C) Coverage map of the 38 kDa band of scuPA K158E, which corresponds to the protease domain of uPA and a fraction of

the linker. (D) Coverage map of the 36 and 38 kDa band of scuPA R156E, which corresponds to the protease domain of uPA. (E) Coverage map of the 38 kDa band of scuPA R156E/K158E, which corresponds to the protease domain of uPA and a fraction of the linker.

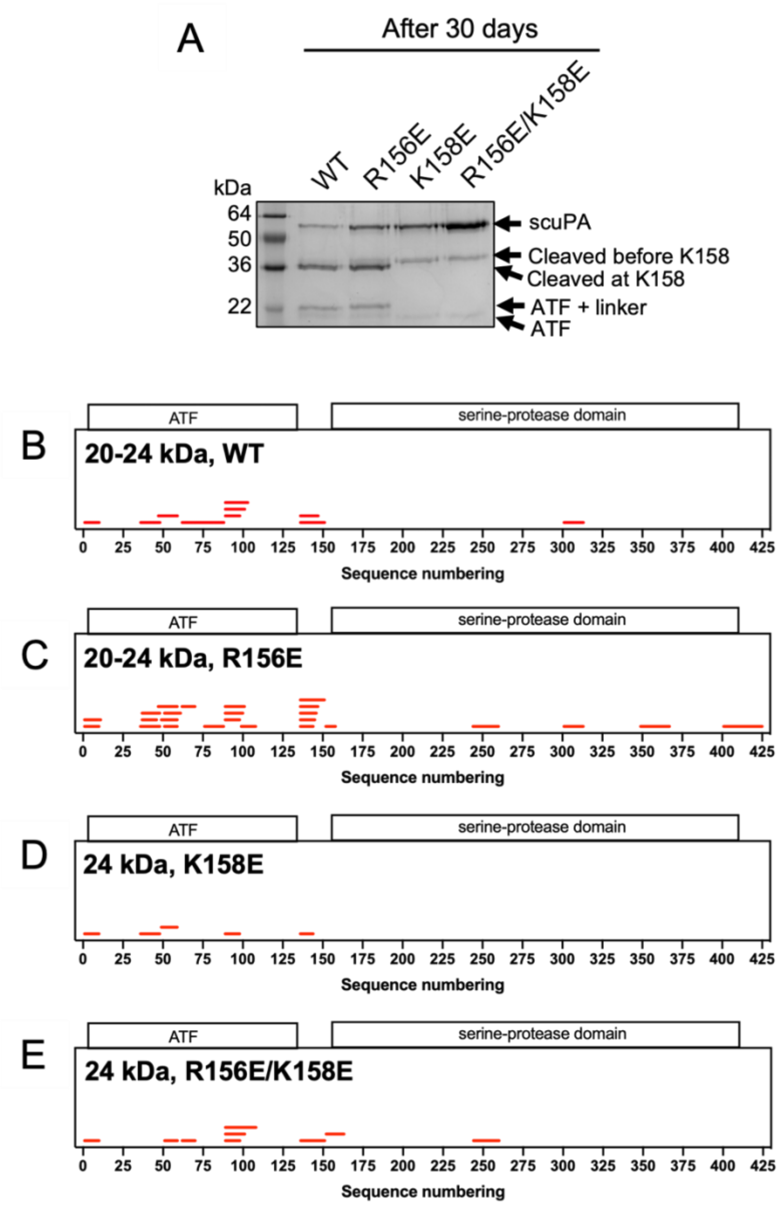

Figure S7. The 20 and 24 kDa bands product of the incubation of scuPA (WT, R156E, K158E and R156E/K158E) at 4˚C for 30 days corresponds to the ATF of uPA. (A) SDS-PAGE under reducing conditions showing the scuPA proteins (WT, R156E, K158E and R156E/K158E) after 30 days of storage at 4˚C. This gel is the same as in Fig. 6E. The bands of this gel were cut, trypsin-digested and sequenced by MS/MS. The peptides detected by MS are shown in the coverage maps as red lines over the sequence they cover. (B) Coverage map of the 20 and 24

kDa band of scuPA WT, which corresponds to the ATF of uPA and a fraction of the linker. (C) Coverage map of the 20 and 24 kDa band of scuPA R156E, which corresponds to the ATF of uPA and a fraction of the linker. (D) Coverage map of the 24 kDa band of scuPA K158E, which corresponds to the ATF of uPA. (E) Coverage map of the 24 kDa band of scuPA R156E/K158E, which corresponds to the ATF of uPA.

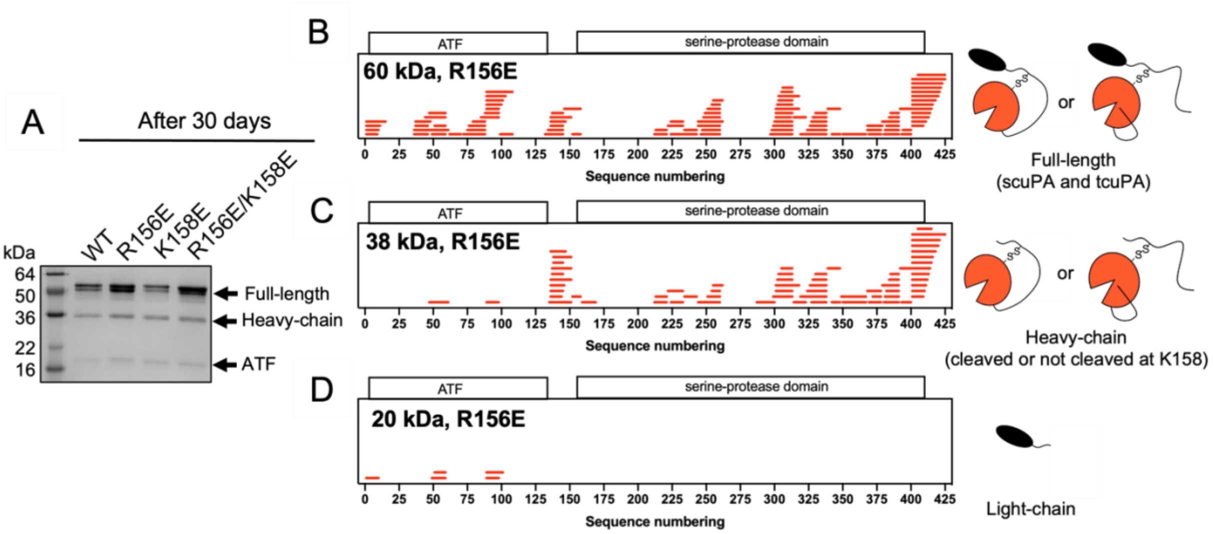

Figure S8. Storage of scuPA R156E at 4˚C for 30 days yields three protein species that were identified by trypsin digestion MS/MS. (A) SDS-PAGE under non-reducing conditions showing the scuPA mutant proteins (WT, R156E, K158E and R156E/K158E) after 30 days of storage at 4˚C.This gel is the same as in Fig. 7F. The bands of this gel were cut, trypsin-digested and sequenced by MS/MS. The peptides detected by MS are shown in the coverage maps as red lines over the sequence they cover. (B) Coverage map of the 60 kDa band that constitutes the full-length scuPA. Note that this protein band could correspond to either the full-length scuPA or the full-length tcuPA as the disulfide bonds are not reduced in this SDS-PAGE. (C) Coverage map of the 36 kDa band that constitutes the heavy-chain of uPA with a portion of the linker. This protein band includes the species cleaved only after K135/K136 and the species cleaved also after K158. (D) Coverage map of the 20 kDa band that corresponds to the ATF.

SUPPORTING TABLES

Table S1. HDX-MS data summary.

**Data Set**	**scuPA WT**	**tcuPA WT**
HDX reaction details	10 mM Na_2HPO_4, 1.8 mM KH_2PO_4, 2.7 mM KCl, 137 mM NaCl (PBS), pH 7.4, 22 °C	
HDX time course (min)	0.5, 1, 2, 5	
HDX control samples	The unstructured ends of the protein were used as maximally deuterated controls	
Back-exchange (mean / IQR)	31 % / 8 %	27 % / 8%
# of Peptides	102	83
Sequence coverage	95.9%	94.4%*
Average peptide length / Redundancy	14.11 / 3.69	14.57 / 3.09
Replicates	2 biological replicates with 3 technical replicates each	
Repeatability	0.0622 (average standard deviation)	0.1135 (average standard deviation)
Significant differences in HDX	0.25 D (99% CI)	
